# Inventing inclusive care norms in the adoption of children with disabilities: coordinating routines, networks and filiation*

**DOI:** 10.1590/1980-220X-REEUSP-2025-0554en

**Published:** 2026-10-05

**Authors:** Luciano Silveira Pacheco de Medeiros, Giovana Calcagno Gomes, Max dos Santos Afonso, Thiago Ferreira Abreu, Jacqueline Flores de Oliveira, Francisco Rosemiro Guimarães Ximenes, Luciano Garcia Lourenção, Clarice Alves Bonow

**Affiliations:** 1 Universidade Federal de Pelotas, Faculdade de Enfermagem, Programa de Pós-Graduação em Enfermagem, Pelotas, RS, Brazil.; 2 Universidade Federal do Rio Grande, Escola de Enfermagem, Programa de Pós-Graduação em Enfermagem, Rio Grande, RS, Brazil.; 3 Universidade Católica de Pelotas, Faculdade de Medicina, Pelotas, RS, Brazil.; 4 Universidade Estadual Vale do Acaraú, Centro de Ciências da Saúde. Sobral, CE, Brazil.

**Keywords:** Adoption, Children with Disability, Primary Health Care, Foster Home Care, Nursing

## Abstract

**Objective:**

To analyze how and why adoptive families of children with disabilities invent and establish norms of inclusive care in their daily lives.

**Method:**

Qualitative study, based on Grounded Theory, carried out in a municipality in southern Brazil, with 11 adoptive families. Individual, face-to-face, semi-structured interviews were conducted and analyzed using constant comparison and open, axial, and selective coding.

**Results:**

The analysis revealed a substantive theory that explains the invention of inclusive care norms as a relational, dynamic, and situated process. The model links causes, context, and intervening conditions to action/interaction strategies guided by an ethics of care, expressed in three movements: coordinating routines, activating networks and advocacy, and recognizing social-affective parentage. These movements produce participation, predictability, comfort, and continuity, sustaining care in contexts marked by institutional fragmentation.

**Conclusion:**

The invention of inclusive care standards expresses the production of situated responses to the child’s needs and institutional limitations. The findings reinforce the importance of care coordination, family involvement, and the role of Nursing in mediating between families and services.

## INTRODUCTION

The adoption of children with disabilities constitutes a privileged field for understanding how social, institutional, and family standards are negotiated and recreated in the daily routine of care. By intertwining adoptive parentage and disability, legal, moral, clinical, educational, and caregiving layers emerge that define those providing care, under what conditions, and with what recognition^(1)^. In heterogeneous and asymmetrical networks, families are frequently called upon to reconcile demands for predictability and continuity with practices that ensure participation and comfort for the child^(2–4)^.

In this context, the family can be understood as a relational system in constant negotiation, constituted by bonds of filiation, care, and belonging, whose organization occurs in the intertwining of daily experiences and institutional demands^(3)^. When assuming the coordination of care in often fragmented settings, families mobilize strategies that support engagement, participation, and continuity of actions, in line with the principles of family-centered care^(2,4)^.

Data from the National Adoption and Foster Care System (SNA) for 2025/2026 shows that Brazil has approximately 34,000 approved applicants for about 5,000 children eligible for adoption^(5)^. However, the health profile remains the main obstacle. It is estimated that around 25% of children and adolescents on the adoption waiting list have some kind of disability or chronic illness, while less than 5% of prospective adoptive parents accept this profile^(6)^. This quantitative disparity demonstrates the vulnerability of these children and adolescents, who face longer waiting times and require adoptive families to undergo a profound regulatory reorganization to fill care gaps.

Although this scenario highlights the centrality of families in sustaining care, the processes by which they produce, negotiate, and stabilize everyday responses remain insufficiently understood. Despite advancements in the literature describing the impacts, barriers, and inequalities associated with disability, explanations regarding the relational and practical mechanisms that allow families to construct, organize, and sustain care in contexts marked by partial, intermittent, or uncoordinated institutional support remain scarce^(7,8)^.

In this study, inclusive care standards are understood as practical and symbolic arrangements that guide daily decisions and routines, produced at the interface between the child’s unique needs and institutional standards. These standards are expressed, in an articulated way, in the organization of routines, the mobilization of networks, and the daily construction of filiation, contributing to the production of participation, predictability, comfort, and continuity in care^(2,4)^.

Given this scenario, the question guiding this study is: how and why do adoptive families of children with disabilities invent and establish standards of inclusive care in their daily lives? Thus, the objective is to analyze how and why adoptive families of children with disabilities invent and stabilize norms of inclusive care.

## METHOD

### Study design

Qualitative study, anchored in *Grounded Theory*, Straussian approach, oriented towards the construction of a substantive theory about the phenomenon described in the thesis “*Inventing standards of inclusive care—coordinating routines, activating networks, and recognizing social-affective parentage—to enhance participation, predictability, comfort, and continuity*”^(9:página)^. The option for *Grounded Theory* is justified by the need to explain relational, dynamic processes situated within contexts of institutional fragmentation, allowing the theoretical model to articulate the conditions, strategies, and consequences of care^(9)^.

The design, centered on social and interactional processes, allows for the understanding of variation, conditions, and chain consequences through constant comparison, theoretical sampling, and cycles of open, axial, and selective coding^(9,10)^, involving the creation of memos and the pursuit of theoretical saturation^(11)^.

From a theoretical-interpretative standpoint, sensitizing concepts—rather than a priori categories for coding—derived from IS and the work by Georges Canguilhem are mobilized to deepen the explanation of the findings without directing the generation of codes^(12–14)^.

### Study Population

The study included 11 participants (six women and five men), predominantly education professionals (university professors, a public school teacher, and a pedagogue), followed by health professionals (two doctors), as well as a retired bricklayer and a homemaker. The family arrangements observed were: adoptions by heterosexual couples (nine occurrences), adoption by a same-sex couple (one occurrence), adoption by a single mother (one occurrence), and intergenerational intrafamilial adoption (one occurrence). In total, 16 children were adopted – counting siblings shared by couples interviewed only once. Regarding the conditions, the most prevalent cases are Cerebral Palsy (CP) with co-occurring Autism Spectrum Disorder (ASD) – levels I to III – and intellectual disability, with co-occurring Attention Deficit Hyperactivity Disorder (ADHD), also co-occurring with ASD.

### Study Location

The study was conducted in a case study municipality in southern Brazil. The care pathways of participating families are articulated within the municipality itself through intersectoral interfaces that include health (Primary Health Care – PHC, specialized outpatient services, rehabilitation centers and pediatric hospitals), education (public and private schools, specialized educational services), social assistance (Social Assistance Reference Center – *CRAS*, Specialized Social Assistance Reference Center – *CREAS*, social protection benefits and services) and the system for guaranteeing rights (Child Protective Services, Public Prosecutor’s Office and Child and Adolescent Court).

This arrangement is central to the child’s participation and continuity of care, but it is often marked by organizational fragmentation and discontinuities, requiring additional coordination work from families, impacting their quality of life and community engagement.

### Selection Criteria

The study included primary caregivers of adoptive families of children with disabilities – congenital or acquired, temporary or permanent, physical, intellectual, physical-intellectual, or multiple – whose condition may have preceded the adoption or been observed/diagnosed during the cohabitation period. Families in the adoption process, in the cohabitation stage, and families under legal guardianship were excluded.

Recruitment of participants began through collaboration with health and education institutions that assist people with disabilities. After a detailed presentation of the project to institutional representatives, support was requested in identifying families that met the eligibility criteria. The initial indications were supplemented by means of the snowball sampling technique^(15)^, in which participants indicated new potential participants.

The theoretical sampling was conducted progressively and guided by the categories that emerged throughout the initial analysis. The initial interviews highlighted the centrality of daily coordination and institutional mediation in care, leading to a deliberate search for variations in family arrangements and contextual conditions capable of challenging and expanding these properties. Thus, distinct configurations were included – heterosexual couples, same-sex couples, single mothers, and intergenerational adoption – as well as different combinations of disabilities, levels of dependency, and institutional trajectories, with the aim of exploring how the phenomenon manifested itself under diverse conditions. The sampling decisions were continually reevaluated in light of the analytical reports, which pointed to gaps in theoretical depth, the need for contrast between cases, or further exploration of dimensions that were still under-explored, in accordance with the principle of constant comparison characteristic of the Straussian Grounded Theory^(9)^.

Initial contact with the nominated candidates was made via *WhatsApp* ^®^ messages, in this presentation, the researcher introduced himself, detailed his academic and professional background, and showed the motivations—both academic and personal—that guided the research. In this initial contact, the objectives and purpose of the study were also presented, establishing clear and transparent communication.

### Data Collection

Data collection was conducted between November 2023 and June 2024. Face-to-face, individual, semi-structured, in-depth interviews were used, with 33 open-ended questions. Four of the interviews were conducted at the participants’ workplaces and seven at their homes. Of the interviews conducted in the homes, only two participants were accompanied: Atlas, accompanied by his adopted son, and Prometheus, accompanied by his mother, an elderly lady of 94 years old. A pilot interview was conducted with one participant, and no adjustments to the interview script were deemed necessary. Given the relatively small study population and the sensitive nature of the topic, this participant was included in the final group of participants. There was no need to repeat any of the interviews.

The script was developed based on the study’s objective and refined through theoretical sampling after preliminary readings, memos, and constant comparisons (case by case and between cases), keeping open questions about: (a) the decision to adopt and reconfiguration of projects; (b) the social, institutional, and symbolic contexts of care; (c) networks and advocacy and interactions with services; (d) coordination of routines (‘living plans’); and (e) perceived effects and transformations.

The interviews were conducted by a professional nurse and doctoral candidate in Nursing, with experience and expertise in qualitative research from the research group they participate in, without any clinical or affective relationship with the participants. The interviews lasted an average of 120–150 minutes. They were recorded and transcribed manually, in semi-verbatim format, with double quality checking and anonymization through pseudonyms. Memoranda and notes—both analytical and descriptive—were produced immediately after each contact. Data collection was concluded due to theoretical saturation when the central categories showed sufficient density in terms of properties, dimensions, and relationships between conditions, strategies, and consequences. From the ninth interview onwards, it was observed that new narratives tended to reiterate variations of already identified properties – especially regarding the coordination of routines, institutional mediation, and the recognition of social-affective parentage – without introducing substantively new categories. The two subsequent interviews served as a robustness test for the model under construction, confirming already established relationships and expanding on empirical examples, but without altering its conceptual architecture. At this point, analytical saturation was considered to have been achieved, understood as the theoretical stability of the central categories^(11)^. There were no refusals.

The data validation process did not include returning the complete transcripts to the participants. Instead, the reliability of the content was ensured through discussion of the researcher’s perceptions and observations with each participant. The strengths and weaknesses observed in the data collection process were analyzed together.

### Data Analysis and Processing

Data analysis and processing were conducted manually. Iterative readings were conducted to segment the data into units of meaning, preserving speech contexts, and coding began with systematic recording in memos that documented analytical decisions and emerging hypotheses^(9,10)^.

Open coding was performed line by line, prioritizing *in vivo* codes and the constant comparison within and between interviews to capture variation, conditions, and consequences of the phenomenon^(10)^. The memos served as traceability devices, guiding the theoretical sampling throughout the data collection process. In axial coding, properties and dimensions of codes were articulated into categories and subcategories, using the conditional/consequential paradigm to model relationships and processes over time^(9)^.

Selective coding integrated the categories around the central phenomenon – “Inventing norms of inclusive care – coordinating routines, activating networks, and recognizing social-affective parentage – to enhance participation, predictability, comfort, and continuity” – resulting in the substantive theoretical model^(9)^. From a theoretical-interpretative standpoint, concepts from Symbolic Interactionism (SI) and Georges Canguilhem’s philosophy were used as analytical lenses to understand the processes of negotiation, meaning-making, and normative production involved in care^(13,14)^. The SI allowed access to the meanings attributed to everyday interactions, while Canguilhem’s perspective grounded the analysis of the “invention of standards” as a vital and normative response of families to environmental adversities.

Throughout the analytical process, in vivo codes such as “making life go on”, “taking care of the paperwork”, and “organizing everything so it doesn’t fall apart” were initially recorded in the open coding as situated coordination actions. In the axial stage, these codes were grouped under the property “intensively coordinating daily life,” articulating with intervening conditions such as institutional fragmentation and bureaucratic demands. Constant comparison between cases revealed that this coordination did not operate in isolation, but was integrated into the mobilization of networks and the recognition of social-affective parentage as a strategy for stabilizing care. In selective coding, these relationships converged to formulate the central phenomenon – “Inventing standards of inclusive care” – understood as the process by which families institute local normative arrangements to support participation, predictability, comfort, and continuity. This movement integrated categories, conditions, and consequences into a procedural theoretical model.

The analytical approach followed the Straussian line of the Grounded Theory, anchored in pragmatism and SI, prioritizing conceptual emergence from empirical data. The categories were constructed through constant comparison and progressive refinement, without the prior application of external theoretical frameworks. These frameworks were used as sensitizing concepts, not categories, a priori, for coding, to deepen the explanation of the findings without directing the generation of codes, preserving the inductive and procedural logic that characterizes the approach adopted^(8,13,14)^.

Data collection was terminated when sufficient theoretical depth was reached to support the analysis guided by the central question. The study rigorously followed the COREQ standards – 32 items for qualitative interview-based studies^(16)^.

### Ethical Aspects

The procedures followed Resolution No. 510/2016 of the National Health Council (*CNS*)^(17)^, with approval from the Research Ethics Committee of the Nursing School of the Universidade Federal de Pelotas – Opinion no. 6,532,379; CAAE 75718623.8.000.5316. Everyone was informed about the partial and final results of the research and that they would be published in technical and scientific circles. All the data supporting the results of this study were published in the article itself.

To preserve the voices of the participants and, simultaneously, guarantee anonymity, the excerpts presented were edited only to remove identifying marks and make minor readability adjustments, without altering the meaning; proper names were replaced according to the anonymization protocol adopted.

## RESULTS

The analysis of the interviews revealed a substantive theory that articulates causes, context, and intervening conditions at the operational level of action/interaction strategies, guided by an ethics of care. The findings showed that adoptive families of children with disabilities build and sustain inclusive care standards through three interdependent movements: coordinating routines (“living plans”), activating networks and advocacy and recognizing social-affective parentage. These actions, when coordinated, produce observable effects of participation, predictability, comfort, and continuity of care.

Data indicate that this process does not occur in a linear fashion. Conversely, sentinel events—such as clinical crises, school disruptions, changes in access to benefits, and transitions in care—trigger reconfigurations in the strategies adopted by families, requiring continuous adjustments to the established standards. It is observed that such events act as critical inflection points, reopening decisions and creating tension in previously established arrangements.

In this context, organizational mediations—related to the functioning of health, education, and social assistance services—modulate the reach of family strategies, potentially facilitating or hindering the continuity of care. Based on the participants’ statements, it is clear that the ability to coordinate between different points in the network directly influences the stability of the standards built in everyday life.

To support the understanding of this process, the theoretical model presented in Figure 1 summarizes the relationships among the identified analytical dimensions. In the model, the action/interaction strategies – coordinating routines (“living plans”), activating networks and advocacy and recognizing socail-affective parentage – are articulated from causes, context and intervening conditions, producing observable consequences: participation, predictability, comfort and continuity of care. The arrows indicate both the main flow and the feedback movements triggered by sentinel events.

**Figure 1:**
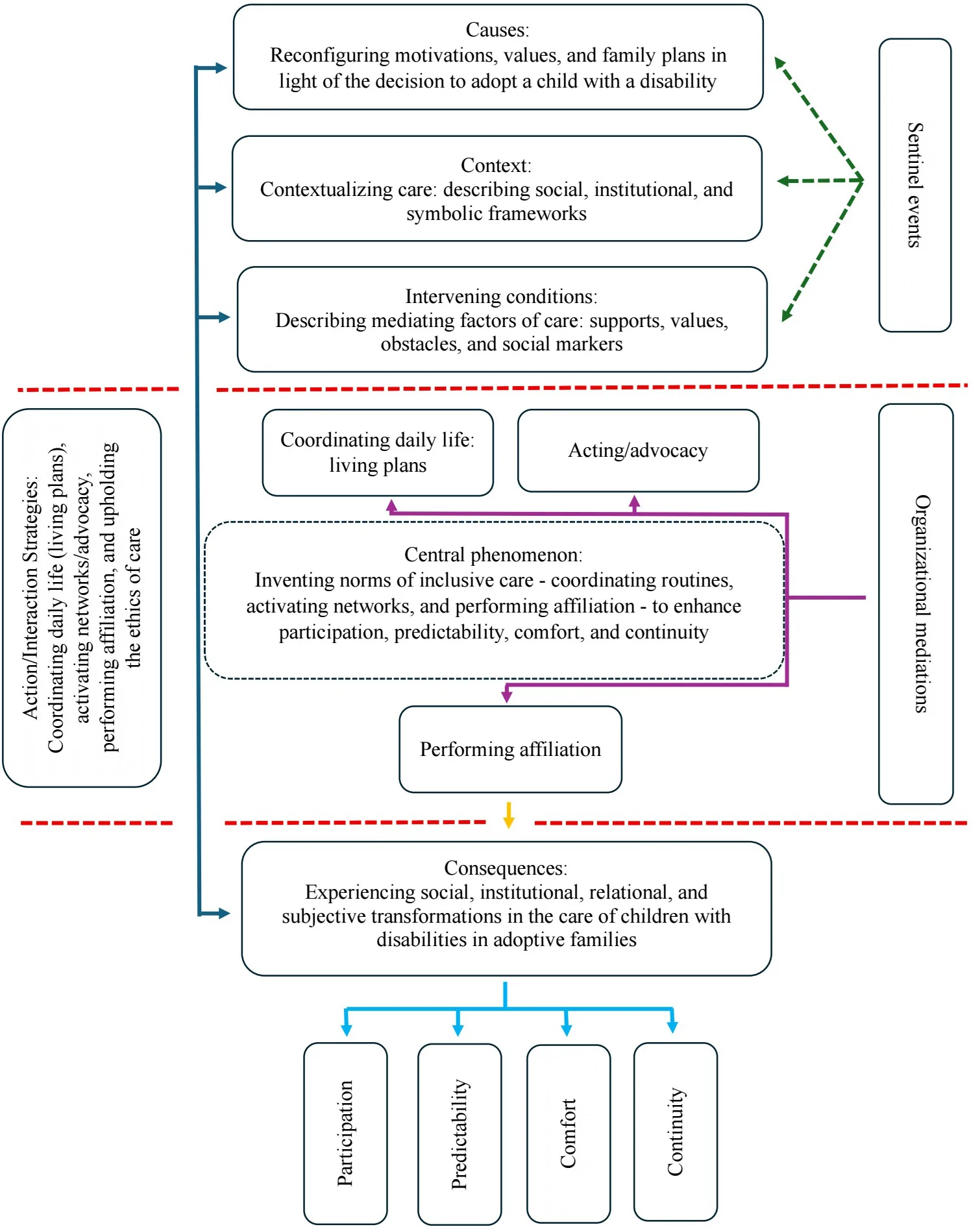
Theoretical model of inclusive care in adoption. Pelotas, RS, Brazil, 2025.

**Source:** own elaboration.

### Starting Point: Decisions, Values and Repositioning (Causes)

Data indicate that the decision to adopt a child with a disability represents a turning point in parenting plans, triggering processes of reconfiguration of values, expectations, and lifestyles. Based on the participants’ statements, it is observed that this decision is not limited to a single event, but rather integrates trajectories marked by reflections on justice, care, and responsibility, which guide the construction of new references for the exercise of parenthood.

*“The decision to adopt came about during the period when we were living in Brasília, due to a professional project of mine, which at the time would require several years away [...].” We were in a phase of emotional and professional stability, and we talked quite frequently about the possibility of expanding our family. The idea of adoption didn’t come from a sudden desire, nor from a specific motivation like infertility or anything like that.” (Athena)*

It is observed that, in this process, families begin to revise expectations related to the child’s development, schooling, and autonomy, replacing previously idealized goals with more flexible horizons adjusted to the concrete conditions of care. This reconfiguration also implies practical changes, such as reorganizing work routines, adapting the home environment, and progressively incorporating knowledge about disability, often built through everyday experience.

*“The house where we live today was given to us by my mother, [...] but it wasn’t prepared to accommodate a child with a disability.” Actually, I don’t think any house is. We discover what we need as our time together unfolds. It’s as if love reveals the needs, one by one. Before our daughter arrived, we did some initial renovations. Since she has reduced mobility, although she can walk short distances with assistance, we knew we would need to adapt her bathroom” (Hestia)*

Based on the narratives, it is clear that these transformations do not occur in a static way, but are continuously updated in response to new diagnostic demands, institutional requirements, and experiences lived in the daily practice of care. This movement supports the development of provisions for the invention of local norms, understood as practical arrangements that allow families to respond to the needs of children in contexts often marked by gaps and discontinuities in services.

### Scenes of Care: Regulatory Layers and Practice Scenarios (Context)

Data indicate that care for adopted children with disabilities takes place across multiple intersectoral settings, involving the home, school, health services, social assistance, and the justice system. Based on the participants’ statements, it is observed that these spaces do not operate in isolation, but constitute a relational field in which formal norms, institutional routines, and daily practices overlap, coexist, and sometimes come into tension.

In this context, school protocols, healthcare network flows, social assistance mechanisms, and legal regulations related to adoption begin to organize access, timelines, and responsibilities in care. However, the data show that such regulations are not entirely stable or sufficient, requiring families to engage in continuous mediation, negotiation, and adaptation to enable participation and continuity of care.

*“At first, the school seemed welcoming. She used all the right words: inclusion, diversity, affection, listening. But in practice, things proved to be much more difficult. Uranus, in the eyes of the professors, was “difficult”. He had difficulty following commands, had meltdowns when his routine was changed, and did not interact with other children in the expected way. And because he didn’t speak, many things were interpreted as disobedience, inattention, or tantrums. From that point on, I understood that my relationship with the school could not be passive.” (Gaia)*

Based on the narratives, it can be observed that institutional responsiveness varies between different services and stages of the care process, producing experiences that oscillate between acceptance and barriers. This variation requires families to develop situated strategies to translate the child’s needs into languages recognized by the services, often mediated by documents such as reports, assessments, and institutional plans.

*“Yes, after adopting Hécate, we started needing several other types of services besides healthcare – mainly in the areas of education and social assistance.” His arrival not only changed our home, it also changed the way we interact with the public support network. We contacted the Municipal Department of Education to understand how his enrollment at the school would work. Since he has cerebral palsy and motor and cognitive limitations, we knew he would be entitled to inclusive education, but we didn’t know how that would happen in practice.” (Demeter)*

*“To begin with, as soon as he came under our care, the people at the courthouse told us to contact CRAS.” That’s where they helped us with the process to get the BPC (Brazilian social security benefit), which is that benefit for people with disabilities. It was paperwork here, reports there, and a visit from the social worker at home. This money is what keeps a lot of things going here today. Without it, we wouldn’t be able to maintain the basics. CRAS also helped with basic food supplies, especially in the beginning, when we were left with the boy without even having diapers to give him. They came here, saw our situation, and brought help. It wasn’t much, but it saved the day at that moment. (Atlas)*

The data show that, within this institutional-family context, home care practices—such as space organization, use of assistive technologies, and the establishment of routines—are continuously interconnected with external demands. When there is misalignment among these spheres, tensions emerge that demand adjustments and reconfigurations in the forms of care, highlighting the dynamic and negotiated nature of the context in which inclusive care standards are produced.

### Forces that Modulate the Achievement (Intervening Conditions)

Data indicate that families’ ability to establish and sustain inclusive care standards does not depend solely on their decisions or strategies, but is strongly modulated by a set of intervening conditions that broaden or restrict the scope of care in daily life. Based on the participants’ statements, it can be observed that these conditions are expressed in material, relational, institutional, and symbolic dimensions, operating in combination throughout the care pathways.

Among these conditions, some act as facilitators, expanding access to resources, strengthening support networks, and promoting continuity of care. Others act as significant barriers, manifesting as insufficient services, fragmented care networks, difficulties in access, family burden, and discontinuity of follow- up care. Data indicate that the way these conditions combine directly influences families’ ability to sustain inclusive care standards over time.

*“Yes, currently our children are in therapy, especially Orion, who has been diagnosed with autism and requires more intensive attention in various areas of development.” From the outset, we recognized the importance of ensuring he receives multidisciplinary and specialized care, capable of providing the stimulation and support he needs to develop with greater autonomy and quality of life. Orion goes to a variety of therapies: follow-up appointments with a neuropediatrician, speech therapist, occupational therapist, and psychologist. Each professional plays an important role, and together they form a technical support network that has gradually contributed to significant improvements in his life.” (Artemis)*

Based on the narratives, it can be observed that the coordination between professionals and services is a central element in shaping care, and can either foster the development of more stable routines or, conversely, produce discontinuities when it is nonexistent or weakened.

*“In fact, he stopped attending therapy sessions shortly after being discharged from the CAPS, shortly after we adopted him” During the period when he was under more intensive care, he had access to different types of therapies, both at the CAPS and at the CER (Specialized Rehabilitation Center). At the CER and CAPS, he received various types of care. He had regular speech therapy sessions for a while. It was clear how much this helped him feel more confident when speaking, and more understood by the people around him. Furthermore, he also received psychological support.” (Prometheus)*

Data also indicate that experiences of embracement or stigma within services influence the empowerment of families, affecting their willingness to seek rights, negotiate with institutions, and sustain care strategies over time. In this sense, access to information, mastery of bureaucratic processes, and the ability to mobilize institutional resources are elements that differentiate more stable trajectories from those marked by greater wear and tear and discontinuity.

Although some participants reported positive experiences with specialized services and support networks, the data also reveal a number of intervening conditions that limit the continuity and effectiveness of care. Among these, the following stand out: a shortage of specialized professionals, an overload of public services, fragmentation of care flows, therapeutic interruptions, difficulties in transportation, and the constant need for bureaucratic mediation on the part of families. These conditions lead to accumulated burnout and transfer responsibilities to caregivers that, in many cases, should be shared by more stable and well- coordinated institutional networks.

*Psychotherapy was the most difficult to obtain. Through the SUS (Brazilian public health system), there were no professionals specialized in childhood autism available. I was referred to CAPSij (Child and Adolescent Psychosocial Care Center), which is overwhelmed and prioritizes acute psychiatric conditions. Urano was evaluated, but because he did not fit the profile of a clinical emergency, he ended up being ‘deregulated’ – that is, not included as a priority. Given this, and seeing that he was showing signs of anxiety, nighttime restlessness, and social isolation, I decided, with great effort, to seek private care. (...) The truth is that there is access to therapies through the SUS, but it is full of obstacles – waiting lists, discontinuity of care, lack of professionals specialized in autism, and even difficulty in getting there, since not all units are accessible or nearby. Persistence is necessary. You need to know the name of the right department, speak to the right employee, and have a copy of all the reports in your bag at all times. And above all: energy is needed. Energy that we don’t always have. (Gaia)*

*“First of all, our lives are very busy and demanding.” Everything revolves around Eduardo. He requires constant care. There’s no way I can just go out and leave him with anyone. He doesn’t speak, he doesn’t walk, you can’t leave him with a neighbor or a grandchild, even if people want to help. We recognize every signal he gives, we know his mannerisms. (...) Helena is in poor health and is currently on disability, but she still helps out in any way she can. When I’m not with Eduardo, I’m trying to get some odd jobs. The children help, but each one has their own life. So our life is here inside, with the boy, with the medications, with the schedules for the clinic, the APAE (Association of Parents and Friends of Exceptional Children), the appointments.” (Atlas)*

It is observed that, when faced with failures in the coordination of services or changes in care conditions, families are frequently led to reconfigure their strategies, adjusting routines, activating new networks, and redefining priorities. These movements demonstrate that the intervening conditions do not act in a static way, but are continuously reinterpreted and confronted in daily life, directly influencing how inclusive care norms are produced and sustained.

### The Tripod of Normative Inventions in Action (Action/interaction Strategies)

Data indicate that the invention and maintenance of inclusive care norms are achieved through three interdependent action/interaction strategies: coordinating routines (“living plans”), activating networks and advocacy and recognizing social-affective parentage. Based on the participants’ statements, it is observed that these strategies do not operate in isolation, but are continuously articulated in daily life, forming a dynamic arrangement that supports the organization of care in response to the child’s needs and the conditions of the context.

*“We returned to our city, with all the care needed for a child with respiratory problems. We lived in a good neighborhood, so the air quality was good, people visited in a controlled manner (...) He did physiotherapy to make up for the time spent in the hospital, did physiotherapy at home (...). When we returned, we went to the person who had the appointment; we knew her husband, we made contact, and she managed to get him into her schedule to see Curetas” (Orpheus)*

*We asked for a week to think it over. We need to talk amongst ourselves. Because it’s a very big change from what we had imagined. So let’s sit down and talk. But at that moment, it was definitive when we saw them.” (Hermes)*

Regarding the coordination of routines, data show that families construct time and activity organization matrices, articulating therapies, school, commutes, leisure time, and rest. It is observed that this coordination involves anticipating demands, developing alternatives in the face of unforeseen events, and continuously adjusting care practices, contributing to the reduction of disruptions and greater predictability in daily life.

*“A very clear example happened yesterday, during an activity at my youngest son’s school.” It was a school party: loud music, lots of people, plenty of sensory stimulation, an unusual time, and the requirement that the children put on presentations for their parents. Among the families present was a friend of ours who has two young children with autism, recently diagnosed. During the activity, these two boys entered a state of disorganization. They were visibly distressed, disoriented, reacting to the overwhelming stimuli imposed by the environment. It was noisy, there were too many people. And there was that mother, completely alone, trying to contain, calm, and protect her children without support, without comfort, without strategies. That affected me deeply. Because we’ve already experienced this with Curetas. We’ve been in that place of despair, of helplessness, of loneliness in the face of our son’s crisis. (Rhea)*

Based on the narratives, it can be observed that institutional responsiveness varies between different services and stages of the care process, producing experiences that oscillate between acceptance and barriers. This variation requires families to develop situated strategies to translate the child’s needs into languages recognized by the services, often mediated by documents such as reports, assessments, and institutional plans.

*“The journey to obtain these therapies began at the health center in our neighborhood.” I took the initial reports (...) and requested referrals through the SUS. That’s when the marathon began. (...) Occupational therapy also came through a referral from the SUS, but not in my city. I managed to get an appointment in a neighboring city, at a partner facility, which treated Leandro for almost six months – until the contract was terminated due to bureaucratic issues between the municipality and the provider. Since then, we have once again become dependent on APAE (...) Psychotherapy was the most difficult to obtain. Through the SUS, there were no professionals specialized in childhood autism available. I was referred to CAPSij (...), but because he didn’t fit that clinical emergency profile, it ended up being ‘deregulated’. (...) The truth is that there is access to therapies through the SUS, but it is full of obstacles – waiting lists, discontinuity of care, lack of professionals specialized in autism, and even difficulty in getting there”.. (Gaia)*

With regard to network and advocacy activation, data indicate that families mobilize formal and informal resources to enable care, involving relatives, professionals, institutions, and collectives. This mobilization includes both establishing support relationships and producing and circulating documents necessary for accessing services and rights.

Participants highlight that building and maintaining these networks requires continuous investment, and is fundamental to expanding the reach of family strategies and reducing institutional barriers. It is observed that the presence of key actors – such as professionals who facilitate access to services – can promote continuity of care.

Regarding the recognition of parentage, data show that families construct, in their daily lives, practices that affirm the child’s belonging beyond the formal legal bond. This strategy is expressed in interactions, narratives, and decisions that position the child as a subject of rights, supporting their inclusion in different care contexts.

*“I do odd jobs as a bricklayer, helper, whatever comes up, because my pension isn’t enough to cover everything.” So the week revolves a lot around that: food, medicine, doctor’s appointments, side jobs, and taking care of Héspero.” (Atlas)*

It is observed that these practices not only organize care, but also produce recognition and symbolic stability, influencing decisions related to the prioritization of activities, the management of institutional demands, and the maintenance of family ties.

Data indicate that these three strategies – coordination of routines, activation of networks, and recognition of parentage – operate in an integrated manner, being continuously adjusted in response to intervening conditions and daily events. This articulation constitutes the operational core of the theoretical model, explaining how families invent and stabilize norms of inclusive care in contexts marked by variability and uncertainty.*.*

### When Norms Materialize: Observable Transformations (Consequences)

Data indicate that when strategies for coordinating routines, activating networks, and recognizing parentage are articulated in a more stable way, they produce observable transformations in care, expressed in four interrelated dimensions: participation, predictability, comfort, and continuity. Based on the participants’ statements, it can be observed that these dimensions do not operate in isolation, but are progressively constructed in the daily lives of families.

*“This experience, more than making us parents of a child with a disability, transformed us as people.” It made us rethink values, revise prejudices, and look at childhood and human diversity with much more depth and humanity.” (Artemis)*

Regarding participation, the data shows greater presence and engagement of children in different contexts, such as school, leisure activities, and social interaction, albeit at varying levels. It is observed that this expansion is associated with the ability of families to adjust routines and negotiate conditions for inclusion in services.

Predictability, in turn, manifests itself in the reduction of uncertainties related to schedules, service flows, and the organization of activities, being built through the anticipation of demands and the consolidation of more stable routines.

*“We started having family moments that included everyone – games, outings, short trips – and that brought us closer together. The day I saw Triton teaching Iapetus how to fit together the pieces of an educational toy was symbolic for me. Without anyone asking, without a camera filming or an audience. It was pure, natural, and full of affection. Today, I have no doubt: they are brothers. “Not just on paper, but in the connection, in the gesture, in the look.” (Cronos)*

In reference to comfort, participants indicate a reduction in situations of sensory and emotional overload, both for the child and the family, associated with adaptations in the environment, communication, and organization of care. Continuity of care is observed in the more consistent coordination between different services and contexts, allowing for less fragmented pathways over time.

*“Today, our support network is well-defined, although not extensive.” We have few, but reliable people around us – and that has been enough [...].” (Athena)*

Based on the narratives, it can be observed that these transformations do not eliminate the difficulties faced by families, but they do indicate the construction of greater stability in care, albeit temporary and subject to reconfiguration.

*“In the beginning, when he came to our house and we took custody of him, we didn’t even know he was entitled to these therapies.” It was the social worker at the courthouse who explained it to us. (...) The doctor requested referrals to a neurologist, a speech therapist, and a physical therapist. They said it might take a while – and it did. To get these therapies, it was a process full of paperwork. I had to bring a medical report, proof of residence, benefit number, photocopies of this, photocopies of that. “There were days when we went to the same place three times just to be able to complete everything.” (Atlas)*

The narratives show that institutional responsiveness varies between different services and stages of the care process, producing experiences that oscillate between acceptance and barriers. This variation requires families to develop situated strategies to translate the child’s needs into languages recognized by the services, often mediated by documents such as reports, assessments, and institutional plans.

Data also indicate the presence of effects associated with this process, such as the intensification of care coordination work and the ongoing need to deal with bureaucratic and institutional demands. At the same time, it is observed that the mobilization of networks and the maintenance of family ties can contribute to the redistribution of responsibilities and the expansion of available support.

*“The relationship between our children is, without a doubt, one of the greatest proofs that love does not depend on biology [...].” It wasn’t just that they ‘accepted’ the brothers. “They truly opened up” (Cronos)*

Finally, data show that the stability of these transformations varies according to the intervening conditions and institutional contexts, being constantly strained by everyday events that demand new adjustments and reconfigurations in the standards constructed by families.

## DISCUSSION

This study proposes a substantive theory that explains how and why adoptive families of children with disabilities invent inclusive care norms to sustain daily life. The results indicate that this process is organized through the articulation of three action/interaction strategies – coordination of routines (“living plans”), activation of networks and advocacy and the recognition of parentage – guided by an ethics of care and modulated by intervening conditions present in institutional and social contexts.

It was observed that these strategies do not operate in isolation, but constitute a dynamic arrangement that allows families to respond to the child’s needs in scenarios marked by fragmented services and discontinuities in care, an aspect widely described in the literature on the care of children with complex conditions and their families^(18–20)^. In this sense, the findings shift the focus away from an analysis centered solely on barriers or impacts, highlighting the procedural mechanisms through which families produce participation, predictability, comfort, and continuity over time^(21,22)^.

Based on the results, it is understood that inclusive care norms are not configured as fixed protocols, but as situated arrangements, built in the interaction between the child’s unique needs and institutional requirements, in line with approaches that highlight the centrality of care coordination and family experiences in health systems^(21,22)^. This dynamic can be interpreted in light of Symbolic Interactionism, highlighting processes of defining situations, negotiating roles, and building joint action in the daily practice of care^(20)^. In dialogue, Canguilhem’s perspective contributes to understanding these norms as expressions of the capacity to establish new operational references in the face of variations from the “normal”^(14)^.

In the field of health, especially in Primary Care, these findings reinforce the strategic role of Nursing in mediating between families and services, in coordinating care, and in building responses that are more sensitive to the concrete needs of children and their caregivers, in line with evidence that points to the importance of integrated and family-centered practices^(4,22)^.

By conceiving inclusive care norms as interactional artifacts, the results highlight the ongoing work of translation, negotiation, and adjustment carried out by families to articulate different care settings, such as home, school, health, and social assistance. It was observed that this process involves not only the practical organization of routines, but also the mediation between institutional expectations and the child’s unique needs, requiring families to play an active role in coordinating care.

The findings indicate that this work is enhanced in contexts marked by fragmented services and a lack of integrated flows, in which families assume the role of connecting points in the network and ensuring continuity of care, an aspect already described in the literature on administrative burdens and user experiences in health systems^(21)^. In this sense, practices such as qualified home visits, the development of Individualized Therapeutic Projects, and longitudinal follow-up, although recognized as central, are still underutilized in Primary Care, limiting the effectiveness of actions and the possibility of building responses more tailored to the family context^(22,23)^.

Based on the results, it can be observed that the norms constructed by families operate as practical devices for stabilizing care, allowing for the alignment of routines, reducing uncertainties, and sustaining the continuity of actions over time. This movement reinforces evidence highlighting the centrality of care coordination and family participation in achieving better health outcomes, especially in more complex contexts^(4,21,22)^.

The results highlight an operational tripod – coordination of routines (“living plans”), activation of networks and advocacy, and the recognition of parentage – as a central mechanism for sustaining care over time. It was observed that these strategies are integrated into the daily lives of families, allowing them to cope with multiple simultaneous demands and respond to often fragmented institutional contexts, in line with evidence that points to the centrality of care coordination and family-centered interventions^(4,7,20,24)^.

Based on the findings, it is identified that the coordination of routines functions as an organizing device for care, reducing disruptions and increasing the predictability of actions, while the activation of networks and advocacy expands access to resources and services, contributing to continuity of care. These results are consistent with studies showing that families of children with complex conditions often assume responsibility for managing care, mediating access and coordinating different services throughout the care pathway^(22)^.

With regard to the recognition of parentage, data indicate that the daily construction of belonging plays a central role in sustaining care, influencing decisions related to the prioritization of activities, the management of institutional demands, and the organization of family routines. This aspect broadens the understanding of care beyond its technical dimension, aligning with approaches that underscore the role of family relationships, bonding, and communication in the production of health care^(4,24)^.

It was also observed that the articulation between these three strategies is guided by an ethics of care that acts as a reference for decision-making in situations of conflict between institutional demands and the child’s needs, as already pointed out in studies that address family-centered care and the need for contextual adjustments in care practices^(4,19)^. In this regard, the operational tripod identified in this study helps to explain how families construct situated responses capable of sustaining participation, predictability, comfort, and continuity in highly complex contexts.

From an interactionist perspective, the results show that the invention of inclusive care standards is built upon continuous processes of defining situations, negotiating roles, and constructing joint action among families, children, and professionals. It was observed that, in the daily routine of caregiving, families interpret institutional demands, adjust expectations, and redefine responsibilities, producing arrangements that allow them to sustain the organization of care in contexts marked by variability and uncertainty.

The findings indicate that these interactions are mediated both by concrete experiences – such as access to services and institutional responsiveness – and by the ability of families to translate their needs into languages recognized by the health, education, and social assistance systems. This movement directly influences who has the legitimacy to participate in decisions and how care is organized over time, in line with studies that highlight the relational and interactional dimension of care coordination^(20)^.

It is also observed that the repetition of experiences – whether successful or difficult – contributes to the stabilization of certain practices, allowing families to consolidate routines, adjust strategies, and sustain norms built in daily life. Within this framework, the results reveal that the predictability of care does not stem solely from institutional protocols, but from the ability to build and maintain agreements situated in the interactions between the different actors involved.

The outcomes imply that Canguilhem’s philosophy contributes to understanding these norms as expressions of the capacity to establish new operational references in the face of variations from the “normal”^(14)^. It was observed that when institutional responses are insufficient to meet the child’s needs, families begin to create their own arrangements—adjusting routines, reorganizing the environment, and redefining priorities—to sustain daily care.

In this regard, the findings indicate that such norms do not constitute deviations from institutional standards, but rather legitimate forms of organizing life, produced at the interface between concrete conditions and the possibilities for action of families. This perspective allows us to understand that continuity of care depends not only on adherence to protocols, but also on the ability to create situated solutions that respond to the specific demands of each context, in line with Canguilhem’s notion of the normativity of living^(14)^.

It is also observed that these processes of normative invention are intensified in scenarios marked by discontinuities in services and inflexible institutional demands, in which families assume an active role in building alternatives to sustain care^(25,26)^. Thus, the results underscore the importance of recognizing these practices not only as adaptive strategies, but also as normative productions that can guide more sensitive and contextualized responses in health services.

This study has limitations that should be considered when interpreting the findings. This is a qualitative investigation, conducted with a limited number of participants and in a single municipality, which does not allow for generalizations, although it favors a deeper understanding of the phenomenon within its context^(27,28,29,30)^. Furthermore, the analysis was constructed from the perspective of caregivers, not directly including the voices of children and adolescents, which may limit the understanding of important dimensions of care. Nonetheless, the use of Grounded Theory and the achievement of theoretical saturation provides analytical robustness to the results, enabling the construction of an explanatory model sensitive to variations and conditions of the phenomenon^(9–11)^.

The results of this study uncover that the invention of inclusive care norms constitutes a relational, dynamic, and situated process, supported by the articulation between family strategies, intervening conditions, and institutional contexts. By making the procedural mechanisms that organize care in daily life explicit, the proposed model contributes to understanding how participation, predictability, comfort, and continuity are produced in contexts marked by fragmentation of services and institutional demands.

## CONCLUSION

This study contributes to the understanding of how and why adoptive families of children with disabilities invent and stabilize norms of inclusive care in daily life. By highlighting the role of routine coordination, network mobilization, and filiation building in sustaining care, the proposed model broadens the analysis of the mechanisms that produce participation, predictability, comfort, and continuity in contexts marked by institutional fragmentation.

In the field of Nursing, the findings redirect the role of the nurse in Primary Health Care towards that of Case Manager and Health Navigator. This professional’s role should extend beyond direct clinical care, focusing on the proactive coordination of intersectoral flows and technical mediation between families and the service network (health, education, and social assistance). Family Nursing, by identifying care gaps and caregiver burden early on, has the potential to reduce the need for families to “invent” rules in isolation, promoting the stabilization of care through integrated therapeutic plans that ensure predictability and continuity of care.

It is recommended that longitudinal, comparative, and implementation studies be developed, as well as investigations that incorporate the perspective of children and adolescents, to deepen the understanding of care processes and the conditions that favor their sustainability.

## Data Availability

The entire dataset supporting the results of this study is available upon request to the corresponding author.
