# Supplementary material for: Inventing inclusive care norms in the adoption of children with disabilities: coordinating routines, networks and filiation
Source: Rev Esc Enferm USP. 2026 Oct 5;60:e20250554. doi: 10.1590/1980-220X-REEUSP-2025-0554en (PMC13640957; doi:10.1590/1980-220X-REEUSP-2025-0554en)
Supplement: Translation

# Translation: Inventando normas de cuidado inclusivo na adoção de crianças com deficiência: coordenando rotinas, redes e filiação^*^

**DOI:** 10.1590/1980-220X-REEUSP-2025-0554pt

**Authors:** Luciano Silveira Pacheco de Medeiros, Giovana Calcagno Gomes, Max dos Santos Afonso, Thiago Ferreira Abreu, Jacqueline Flores de Oliveira, Francisco Rosemiro Guimarães Ximenes, Luciano Garcia Lourenção, Clarice Alves Bonow

**Affiliations:** 1 Universidade Federal de Pelotas, Faculdade de Enfermagem, Programa de Pós-Graduação em Enfermagem, Pelotas, RS, Brasil.; 2 Universidade Federal do Rio Grande, Escola de Enfermagem, Programa de Pós-Graduação em Enfermagem, Rio Grande, RS, Brasil.; 3 Universidade Católica de Pelotas, Faculdade de Medicina, Pelotas, RS, Brasil.; 4 Universidade Estadual Vale do Acaraú, Centro de Ciências da Saúde. Sobral, CE, Brasil.

**Keywords:** Adoção, Crianças com Deficiência, Atenção Primária à Saúde, Cuidados no Lar de Adoção, Enfermagem.

## Abstract

**Objetivo:**

Analisar como e por que famílias adotivas de crianças com deficiência inventam e estabilizam normas de cuidado inclusivo no cotidiano.

**Método:**

Estudo qualitativo, fundamentado na Grounded Theory, realizado em um município do sul do Brasil, com 11 famílias adotivas. Foram realizadas entrevistas individuais, presenciais e semiestruturadas, analisadas por comparação constante e codificação aberta, axial e seletiva.

**Resultados:**

A análise revelou uma teoria substantiva que explica a invenção de normas de cuidado inclusivo como processo relacional, dinâmico e situado. O modelo articula causas, contexto e condições intervenientes a estratégias de ação/interação orientadas por uma ética do cuidado, expressas em três movimentos: coordenar rotinas, acionar redes e advocacy e performar a filiação. Esses movimentos produzem participação, previsibilidade, conforto e continuidade, sustentando o cuidado em contextos marcados por fragmentação institucional.

**Considerações finais:**

A invenção de normas de cuidado inclusivo expressa a produção de respostas situadas frente às demandas da criança e às limitações institucionais. Os achados reforçam a importância da coordenação do cuidado, da participação familiar e da atuação da Enfermagem na mediação entre famílias e serviços.

## INTRODUÇÃO

A adoção de crianças com deficiência constitui um campo privilegiado para compreender como normas sociais, institucionais e familiares são negociadas e recriadas no cotidiano do cuidado. Ao entrecruzar filiação adotiva e deficiência, emergem camadas jurídicas, morais, clínicas, escolares e assistenciais que definem quem cuida, em que condições e com que reconhecimento^(1)^. Em redes heterogêneas e assimétricas, as famílias são frequentemente convocadas a articular exigências de previsibilidade e continuidade com práticas que assegurem participação e conforto à criança^(2–4)^.

Nesse contexto, a família pode ser compreendida como um sistema relacional em constante negociação, constituído por vínculos de filiação, cuidado e pertencimento, cuja organização se dá no entrelaçamento entre experiências cotidianas e exigências institucionais^(3)^. Ao assumir a coordenação do cuidado em cenários frequentemente fragmentados, as famílias mobilizam estratégias que sustentam o engajamento, a participação e a continuidade das ações, em consonância com princípios do cuidado centrado na família^(2,4)^.

Dados do Sistema Nacional de Adoção e Acolhimento (SNA) de 2025/2026 evidenciam que o Brasil possui cerca de 34.000 pretendentes habilitados para aproximadamente 5.000 crianças aptas à adoção^(5)^. No entanto, o perfil de saúde permanece como o principal obstáculo. Estima-se que cerca de 25% das crianças e adolescentes na fila de adoção possuem alguma deficiência ou doença crônica, enquanto menos de 5% dos pretendentes aceitam esse perfil^(6)^. Esse descompasso quantitativo mostra a vulnerabilidade dessas crianças e adolescentes, que enfrentam tempos de espera superiores e demandam das famílias adotivas uma reorganização normativa profunda para suprir lacunas assistenciais.

Embora esse cenário evidencie a centralidade das famílias na sustentação do cuidado, os processos pelos quais elas produzem, negociam e estabilizam respostas cotidianas permanecem insuficientemente compreendidos. Apesar do avanço da literatura na descrição de impactos, barreiras e desigualdades associados à deficiência, ainda são escassas as explicações sobre os mecanismos relacionais e práticos que permitem às famílias construir, organizar e sustentar o cuidado em contextos marcados por suportes institucionais parciais, intermitentes ou descoordenados^(7,8)^.

Neste estudo, compreendem-se normas de cuidado inclusivo como arranjos práticos e simbólicos que orientam decisões e rotinas no cotidiano, produzidos na interface entre necessidades singulares da criança e padrões institucionais. Essas normas expressam-se, de forma articulada, na organização das rotinas, na mobilização de redes e na construção cotidiana da filiação, contribuindo para a produção de participação, previsibilidade, conforto e continuidade no cuidado^(2,4)^.

Diante desse panorama, a pergunta que orienta este estudo é: como e por que famílias adotivas de crianças com deficiência inventam e estabilizam normas de cuidado inclusivo no cotidiano? Assim, o objetivo é analisar como e por que famílias adotivas de crianças com deficiência inventam e estabilizam normas de cuidado inclusivo.

## MÉTODO

### Desenho do Estudo

Estudo qualitativo, ancorado na *Grounded Theory*, vertente straussiana, orientado à construção de teoria substantiva sobre o fenômeno da tese “*Inventando normas de cuidado inclusivo – coordenando rotinas, acionando redes e performando filiação – para elevar participação, previsibilidade, conforto e continuidade*”^(9:página)^. A opção pela *Grounded Theory* justifica-se pela necessidade de explicar processos relacionais, dinâmicos e situados em contextos de fragmentação institucional, permitindo que o modelo teórico articule condições, estratégias e consequências do cuidado^(9)^.

O delineamento, centrado em processos sociais e interacionais, permite apreender variação, condições e consequências em cadeia por meio de comparação constante, amostragem teórica e ciclos de codificação aberta, axial e seletiva^(9,10)^, com elaboração de memos e busca de saturação teórica^(11)^.

No plano teórico-interpretativo, mobilizam-se conceitos sensibilizadores – e não categorias a priori para codificação – provenientes do IS e da obra de Georges Canguilhem, a fim de aprofundar a explicação dos achados sem dirigir a geração dos códigos^(12–14)^.

### População do Estudo

O estudo contou com 11 participantes (seis mulheres e cinco homens), com predomínio de profissionais da educação (professoras e professores universitários, professora da rede pública e pedagoga), seguidos de profissionais da saúde (dois médicos), além de um pedreiro aposentado e uma dona de casa. Os arranjos familiares observados foram: adoções por casais heteroafetivos (nove ocorrências), adoção por casal homoafetivo (uma ocorrência), adoção por mãe solo (uma ocorrência) e adoção intrafamiliar intergeracional (uma ocorrência). Ao todo, foram 16 crianças adotadas – contabilizando fratrias compartilhadas por casais entrevistados apenas uma vez. Quanto às condições, prevalecem os casos de Paralisia Cerebral (PC) com coocorrência de Transtorno do Espectro Autista (TEA) – níveis I a III – e deficiência intelectual, com coocorrência de Transtorno de Déficit de Atenção e Hiperatividade (TDAH), também em coocorrência com TEA*.*

### Local do Estudo

O estudo foi desenvolvido em um município-cenário do sul do Brasil. As trajetórias de cuidado das famílias participantes articulam-se, no próprio município, por interfaces intersetoriais que incluem saúde (Atenção Primária à Saúde – APS, serviços especializados ambulatoriais, centros de reabilitação e hospitais pediátricos), educação (escolas públicas e privadas, atendimento educacional especializado), assistência social (Centro de Referência de Assistência Social – CRAS, Centro de Referência Especializado de Assistência Social – CREAS, benefícios e serviços de proteção social) e o sistema de garantia de direitos (Conselho Tutelar, Ministério Público e Vara da Infância e Juventude).

Esse arranjo é central para participação e continuidade do cuidado da criança, mas é frequentemente marcado por fragmentação organizacional e descontinuidades, demandando trabalho adicional de coordenação por parte das famílias, repercutindo em sua qualidade de vida e engajamento comunitário.

### Critério de Seleção

Incluíram-se cuidadores(as) principais de famílias adotivas de crianças com deficiência – congênita ou adquirida, transitória ou permanente, física, intelectual, físico-intelectual ou múltipla – cuja condição podia anteceder a adoção ou ser observada/diagnosticada durante o estágio de convivência. Excluíram-se famílias em processo de adoção, no estágio de convivência, e famílias em situação de tutela judicial.

Iniciou-se o recrutamento de participantes por meio da colaboração de instituições de saúde e educação que assistem pessoas com deficiência. Após a apresentação detalhada do projeto aos responsáveis institucionais, foi solicitado o apoio para a identificação de famílias que atendessem aos critérios de elegibilidade. As indicações iniciais foram complementadas por meio da técnica de amostragem em bola de neve (*snowball*)^(15)^, em que os participantes indicavam novos potenciais participantes.

A amostragem teórica foi conduzida de modo progressivo e orientada pelas categorias emergentes ao longo da análise inicial. As primeiras entrevistas evidenciaram a centralidade do trabalho de coordenação cotidiana e da mediação institucional no cuidado, o que levou à busca deliberada por variação em arranjos familiares e condições contextuais capazes de tensionar e expandir essas propriedades. Assim, foram incluídas configurações distintas – casais heteroafetivos, casal homoafetivo, mãe solo e adoção intergeracional – bem como diferentes combinações de deficiências, níveis de dependência e trajetórias institucionais, com o objetivo de explorar como o fenômeno se manifestava sob condições diversas. As decisões amostrais foram continuamente reavaliadas à luz dos memorandos analíticos, que apontavam lacunas de densidade teórica, necessidade de contraste entre casos ou aprofundamento de dimensões ainda pouco exploradas, em consonância com o princípio da comparação constante característico da *Grounded Theory straussiana* ^(9)^.

O contato inicial com os candidatos indicados foi realizado por mensagens de *WhatsApp* ^®^, na qual o pesquisador se apresentou, detalhou sua trajetória acadêmica e profissional, e expôs as motivações – tanto acadêmicas quanto pessoais – que orientaram a pesquisa. Neste primeiro contato, também foram apresentados os objetivos e a finalidade do estudo, estabelecendo uma comunicação clara e transparente.

### Coleta dos Dados

A coleta foi conduzida entre novembro de 2023 e junho de 2024. Utilizaram-se entrevistas presenciais, individuais, semiestruturadas e em profundidade, com 33 perguntas abertas. Quatro das entrevistas foram realizadas nos locais de trabalho dos participantes e sete em suas residências. Das entrevistas realizadas nas residências, apenas dois participantes estavam acompanhados: Atlas, acompanhado do seu filho adotivo, e Prometeu, acompanhado da sua mãe, uma senhora idosa de 94 anos. Realizou-se entrevista-piloto com um participante e não se observou necessidade de ajustes no roteiro de perguntas. Por se tratar de uma população de estudo bastante pequena e de uma temática sensível, esse participante foi incluído ao grupo final de participantes. Não houve a necessidade de repetir nenhuma das entrevistas.

O roteiro foi elaborado a partir do objetivo do estudo e aprimorado por amostragem teórica após leituras preliminares, memos e comparações constantes (do caso a caso e entre casos), mantendo perguntas abertas sobre: (a) decisão de adotar e reconfiguração de projetos; (b) contextos sociais, institucionais e simbólicos do cuidado; (c) redes e *advocacy* e interações com serviços; (d) coordenação de rotinas (“planos vivos”); e (e) efeitos e transformações percebidas.

As entrevistas foram conduzidas por profissional enfermeiro e doutorando em Enfermagem, com experiência e expertise em pesquisa qualitativa pelo grupo de pesquisa que participa, sem vínculo assistencial e afetivo com os participantes. As entrevistas tiveram duração média de 120-150 minutos. Foram gravadas e transcritas, manualmente, em formato semi-verbatim, com dupla checagem de qualidade e anonimização por pseudônimos. Memorandos e notas – analíticas e descritivas – foram produzidos imediatamente após cada contato. A coleta foi encerrada por saturação teórica quando as categorias centrais apresentaram densidade suficiente em termos de propriedades, dimensões e relações entre condições, estratégias e consequências. A partir da nona entrevista, observou-se que novas narrativas tendiam a reiterar variações de propriedades já identificadas – especialmente no que se refere à coordenação de rotinas, mediação institucional e performação da filiação – sem introduzir categorias substantivamente novas. As duas entrevistas subsequentes funcionaram como teste de robustez do modelo em construção, confirmando relações já estabelecidas e ampliando exemplos empíricos, mas sem alterar sua arquitetura conceitual. Nesse ponto, considerou-se alcançada a saturação analítica, entendida como estabilidade teórica das categorias centrais^(11)^. Não houve recusas.

O processo de validação dos dados não incluiu a devolução das transcrições completas aos participantes. Em vez disso, a fidedignidade do conteúdo foi assegurada por meio da discussão das percepções e observações do pesquisador com cada participante. Foram analisadas, em conjunto, as forças e as fragilidades observadas no processo de coleta de dados.

### Análise e Tratamento dos Dados

A análise e o tratamento dos dados foram conduzidos manualmente. Procedeu-se a leituras iterativas para segmentação em unidades de sentido, preservando contextos de fala, e iniciou-se a codificação com registro sistemático em memorandos que documentaram decisões analíticas e hipóteses emergentes^(9,10)^.

A codificação aberta foi realizada linha a linha, privilegiando códigos *in vivo* e a comparação constante dentro e entre entrevistas para captar variação, condições e consequências do fenômeno^(10)^. Os memorandos funcionaram como dispositivos de rastreabilidade, orientando a amostragem teórica ao longo da coleta. Na codificação axial, articularam-se propriedades e dimensões dos códigos em categorias e subcategorias, utilizando o paradigma condicional/consequencial para modelar relações e processos ao longo do tempo^(9)^.

A codificação seletiva integrou as categorias em torno do fenômeno central – “Inventando normas de cuidado inclusivo – coordenando rotinas, acionando redes e performando filiação – para elevar participação, previsibilidade, conforto e continuidade” -, resultando no modelo teórico substantivo^(9)^. No plano teórico-interpretativo, mobilizaram-se conceitos do Interacionismo Simbólico (IS) e da filosofia de Georges Canguilhem como lentes analíticas para compreender os processos de negociação, construção de sentidos e produção normativa implicados no cuidado^(13,14)^. O IS permitiu acessar os significados atribuídos às interações cotidianas, enquanto a perspectiva de Canguilhem fundamentou a análise da “invenção de normas” como uma resposta vital e normativa das famílias frente às adversidades do meio.

Ao longo do processo analítico, códigos in vivo como “fazendo a vida caminhar”, “dando conta da papelada” e “organizando tudo para não desandar” foram inicialmente registrados na codificação aberta como ações situadas de coordenação. Na etapa axial, tais códigos foram agrupados sob a propriedade “coordenando intensivamente o cotidiano”, articulando-se a condições intervenientes como fragmentação institucional e exigências burocráticas. A comparação constante entre casos revelou que essa coordenação não operava isoladamente, mas se integrava à mobilização de redes e à performação da filiação como estratégia de estabilização do cuidado. Na codificação seletiva, essas relações convergiram para a formulação do fenômeno central – “Inventando normas de cuidado inclusivo” – entendido como processo pelo qual as famílias instituem arranjos normativos locais para sustentar participação, previsibilidade, conforto e continuidade. Esse movimento integrou categorias, condições e consequências em um modelo teórico processual.

A condução analítica seguiu a vertente straussiana da *Grounded Theory*, ancorada no pragmatismo e no IS, privilegiando a emergência conceitual a partir dos dados empíricos. As categorias foram construídas por meio de comparação constante e refinamento progressivo, sem aplicação prévia de estruturas teóricas externas. Tais referenciais foram utilizados como conceitos sensibilizadores e não categorias, a priori, para codificação, a fim de aprofundar a explicação dos achados sem dirigir a geração dos códigos, preservando a lógica indutiva e processual que caracteriza a abordagem adotada^(8,13,14)^.

A coleta terminou ao atingir densidade teórica suficiente para sustentar a análise orientada pela questão central. O estudo seguiu rigorosamente os padrões do COREQ – 32 itens para estudos qualitativos baseados em entrevistas^(16)^.

### Aspectos Éticos

Os procedimentos seguiram a Resolução do Conselho Nacional de Saúde (CNS) nº 510/2016^(17)^, com aprovação do Comitê de Ética em Pesquisa da Faculdade de Enfermagem da Universidade Federal de Pelotas – Parecer n. 6.532.379; CAAE 75718623.8.000.5316. Todos(as) foram informados(as) sobre os resultados parciais e finais da pesquisa e de que estes seriam divulgados em meios técnico-científicos. Todo o conjunto de dados que dá suporte aos resultados deste estudo foi publicado no próprio artigo.

Para preservar a voz dos(as) participantes e, simultaneamente, garantir a desidentificação, os excertos apresentados foram editados apenas para supressão de marcas identificadoras e pequenos ajustes de legibilidade, sem alteração de sentido; nomes próprios foram substituídos conforme o protocolo de anonimização adotado.

## RESULTADOS

A análise das entrevistas revelou uma teoria substantiva que articula causas, contexto e condições intervenientes ao nível operativo das estratégias de ação/interação, orientadas por uma ética do cuidado. Os achados evidenciaram que as famílias adotivas de crianças com deficiência constroem e sustentam normas de cuidado inclusivo por meio de três movimentos interdependentes: coordenar rotinas (“planos vivos”), acionar redes e *advocacy* e performar a filiação. Esses movimentos, quando articulados, produzem efeitos observáveis de participação, previsibilidade, conforto e continuidade do cuidado.

Os dados indicam que esse processo não ocorre de forma linear. Ao contrário, eventos-sentinela – como crises clínicas, rupturas escolares, mudanças no acesso a benefícios e transições no cuidado – desencadeiam reconfigurações nas estratégias adotadas pelas famílias, exigindo ajustes contínuos nas normas construídas. Observa-se que tais eventos operam como pontos críticos de inflexão, reabrindo decisões e tensionando arranjos previamente estabilizados.

Nesse contexto, mediações organizacionais – relacionadas ao funcionamento dos serviços de saúde, educação e assistência social – modulam o alcance das estratégias familiares, podendo facilitar ou dificultar a continuidade do cuidado. A partir das falas dos participantes, identifica-se que a capacidade de articulação entre diferentes pontos da rede influencia diretamente a estabilidade das normas construídas no cotidiano.

Para apoiar a compreensão desse processo, apresenta-se o modelo teórico na Figura 1, que sintetiza as relações entre as dimensões analíticas identificadas. No modelo, as estratégias de ação/interação – coordenar rotinas (“planos vivos”), acionar redes e *advocacy* e performar a filiação – articulam-se a partir de causas, contexto e condições intervenientes, produzindo consequências observáveis: participação, previsibilidade, conforto e continuidade do cuidado. As setas indicam tanto o fluxo principal quanto os movimentos de retroalimentação acionados por eventos-sentinela.

**Figura 1:** Modelo teórico do cuidado inclusivo na adoção. Pelotas, RS, Brasil, 2025.

**Fonte:** elaboração própria.

### Ponto de Partida: Decisões, Valores e Reposicionamentos (Causas)

Os dados indicam que a decisão de adotar uma criança com deficiência configura-se como um ponto de inflexão nos projetos de parentalidade, mobilizando processos de reconfiguração de valores, expectativas e modos de vida. A partir das falas dos participantes, observa-se que essa decisão não se reduz a um evento pontual, mas integra trajetórias marcadas por reflexões sobre justiça, cuidado e responsabilidade, que orientam a construção de novas referências para o exercício da parentalidade.

*“A decisão pela adoção surgiu durante o período em que estávamos morando em Brasília, por conta de um projeto profissional meu, que na época demandaria alguns anos fora [...]. Estávamos numa fase de estabilidade emocional e profissional, e conversávamos com alguma frequência sobre a possibilidade de ampliar a família. A ideia da adoção não veio de um desejo repentino, nem de uma motivação específica como infertilidade ou algo do tipo.” (Atena)*

Observa-se que, nesse processo, as famílias passam a revisar expectativas relacionadas ao desenvolvimento, à escolarização e à autonomia da criança, substituindo metas previamente idealizadas por horizontes mais flexíveis e ajustados às condições concretas do cuidado. Essa reconfiguração implica também mudanças práticas, como reorganização de rotinas de trabalho, adaptações no espaço doméstico e incorporação progressiva de saberes sobre deficiência, frequentemente construídos na experiência cotidiana.

*“A casa onde a gente mora hoje foi cedida pela minha mãe, [...] mas que não estava preparada para acolher uma criança com deficiência. Na verdade, acho que nenhuma casa está. A gente vai descobrindo o que precisa à medida que a convivência vai se desenrolando. É como se o amor fosse revelando as necessidades, uma a uma. Antes da chegada da nossa filha, fizemos uma reforma inicial. Como ela tem mobilidade reduzida, embora consiga caminhar curtas distâncias com ajuda, sabíamos que precisaríamos adaptar o banheiro dela.” (Héstia)*

A partir das narrativas, identifica-se que essas transformações não ocorrem de forma estática, sendo continuamente reatualizadas diante de novas demandas diagnósticas, exigências institucionais e experiências vividas no cotidiano do cuidado. Esse movimento sustenta a construção de disposições para a invenção de normas locais, entendidas como arranjos práticos que permitem às famílias responder às necessidades da criança em contextos frequentemente marcados por lacunas e descontinuidades nos serviços.

### Cenas do Cuidado: Camadas Regulatórias e Cenários de Prática (Contexto)

Os dados indicam que o cuidado às crianças adotadas com deficiência configura-se em múltiplos cenários intersetoriais, envolvendo domicílio, escola, serviços de saúde, assistência social e sistema de justiça. A partir das falas dos participantes, observa-se que esses espaços não operam de forma isolada, mas constituem um campo relacional no qual normas formais, rotinas institucionais e práticas cotidianas sobrepõem-se, coexistem e, por vezes, entram em tensão.

Nesse contexto, protocolos escolares, fluxos da rede de saúde, dispositivos socioassistenciais e normativas jurídicas relacionadas à adoção passam a organizar o acesso, os tempos e as responsabilidades no cuidado. No entanto, os dados evidenciam que tais normativas não são plenamente estáveis ou suficientes, exigindo das famílias um trabalho contínuo de mediação, negociação e adaptação para viabilizar a participação e a continuidade do cuidado.

*“No início, a escola parecia acolhedora. Usava todas as palavras corretas: inclusão, diversidade, afeto, escuta. Mas na prática as coisas se mostraram bem mais difíceis. O Urano, aos olhos dos professores, era “difícil”. Não seguia comandos com facilidade, tinha crises quando a rotina era alterada, não interagia da forma esperada com as outras crianças. E, como não falava, muita coisa era interpretada como desobediência, como desatenção, como birra. A partir dali, entendi que minha relação com a escola não poderia ser passiva.” (Gaia)*

Observa-se, a partir das narrativas, que a responsividade institucional varia entre os diferentes serviços e momentos do percurso de cuidado, produzindo experiências que oscilam entre acolhimento e barreiras. Essa variação exige das famílias a construção de estratégias situadas para traduzir as necessidades da criança em linguagens reconhecidas pelos serviços, frequentemente mediadas por documentos como laudos, relatórios e planos institucionais.

*“Sim, depois da adoção do Hécate, passamos a precisar de vários outros tipos de serviço além da saúde – principalmente nas áreas da educação e da assistência social. A chegada dele não mudou só a nossa casa, mudou também a forma como nos relacionamos com a rede pública de apoio. Procuramos a Secretaria Municipal de Educação pra entender como funcionaria a matrícula dele na escola. Como ele tem paralisia cerebral e limitações motoras e cognitivas, sabíamos que ele teria direito à educação inclusiva, mas não sabíamos como isso aconteceria na prática.” (Deméter)*

*“Para começar, logo que ele passou a ficar sob nossa guarda, o pessoal do fórum já falou para gente procurar o CRAS. Foi lá que nos ajudaram com o processo para conseguir o BPC* [Benefício de Prestação Continuada]*, que é aquele benefício para pessoa com deficiência. Foi papel para cá, laudo para lá, visita da assistente social em casa. Esse dinheiro hoje é o que segura muita coisa aqui. Sem ele, a gente não conseguiria manter o básico. O CRAS também ajudou com cesta básica, principalmente no começo, quando a gente ficou com o menino sem ter nem fralda para dar. Vieram aqui, viram nossa situação, e trouxeram ajuda. Não foi muito, mas naquela hora salvou.” (Atlas)*

Os dados mostram que, nesse campo institucional-familiar, as práticas de cuidado no domicílio – como organização do espaço, uso de tecnologias assistivas e construção de rotinas – articulam-se continuamente com exigências externas. Quando há desalinhamento entre essas esferas, emergem tensões que demandam ajustes e reconfigurações nas formas de cuidado, evidenciando o caráter dinâmico e negociado do contexto em que as normas de cuidado inclusivo são produzidas.

### Forças que Modulam o Alcance (Condições intervenientes)

Os dados indicam que a capacidade das famílias de instituir e sustentar normas de cuidado inclusivo não depende apenas de suas decisões ou estratégias, sendo fortemente modulada por um conjunto de condições intervenientes que ampliam ou restringem o alcance do cuidado no cotidiano. A partir das falas dos participantes, observa-se que tais condições se expressam em dimensões materiais, relacionais, institucionais e simbólicas, operando de forma combinada ao longo das trajetórias de cuidado.

Entre essas condições, algumas atuam como facilitadoras, ampliando o acesso a recursos, fortalecendo redes de apoio e favorecendo a continuidade do cuidado. Outras operam como barreiras importantes, expressando-se na insuficiência de serviços, na fragmentação das redes assistenciais, nas dificuldades de acesso, na sobrecarga familiar e na descontinuidade dos acompanhamentos. Os dados indicam que a forma como essas condições se combinam influencia diretamente a capacidade das famílias de sustentar normas de cuidado inclusivo ao longo do tempo.

*“Sim, atualmente nossos filhos fazem terapias, principalmente o Órion, que tem diagnóstico de autismo e exige uma atenção mais intensiva em diversas áreas do desenvolvimento. Desde o início, percebemos a importância de garantir a ele um acompanhamento multidisciplinar e especializado, capaz de oferecer os estímulos e apoios que ele precisa para se desenvolver com mais autonomia e qualidade de vida. O Órion faz diversas terapias: acompanhamento com neuropediatra, fonoaudióloga, terapeuta ocupacional e psicóloga. Cada profissional tem um papel importante, e juntos formam uma rede de suporte técnico que, aos poucos, tem contribuído para avanços significativos na vida dele.” (Ártemis)*

Observa-se, a partir das narrativas, que a articulação entre profissionais e serviços constitui um elemento central na modulação do cuidado, podendo favorecer a construção de rotinas mais estáveis ou, ao contrário, produzir descontinuidades quando inexistente ou fragilizada.

*“Na verdade, ele parou com os acompanhamentos terapêuticos logo depois que recebeu alta do CAPS, pouco tempo depois que foi adotado por nós. Durante o período em que esteve sob acompanhamento mais intenso, ele teve acesso a diferentes tipos de terapias, tanto no CAPS quanto no CER (Centro Especializado de Reabilitação). No CER e no CAPS, ele passou por vários tipos de atendimentos. Ele teve sessões regulares de fonoaudiologia por um tempo. Era visível o quanto isso ajudava ele a se sentir mais seguro ao falar, mais compreendido pelas pessoas ao seu redor. Além disso, ele também teve acompanhamento psicológico.” (Prometeu)*

Os dados indicam ainda que experiências de acolhimento ou estigma nos serviços influenciam o protagonismo das famílias, afetando sua disposição para buscar direitos, negociar com instituições e sustentar estratégias de cuidado ao longo do tempo. Nesse sentido, o acesso a informações, o domínio de processos burocráticos e a capacidade de mobilizar recursos institucionais configuram-se como elementos que diferenciam trajetórias mais estáveis daquelas marcadas por maior desgaste e descontinuidade.

Embora parte dos participantes tenha relatado experiências positivas com serviços especializados e redes de apoio, os dados também evidenciam um conjunto de condições intervenientes dificultadoras que limitam a continuidade e a efetividade do cuidado. Entre elas destacam-se a insuficiência de profissionais especializados, a sobrecarga dos serviços públicos, a fragmentação dos fluxos assistenciais, as interrupções terapêuticas, as dificuldades de deslocamento e a necessidade constante de mediação burocrática por parte das famílias. Essas condições produzem desgaste acumulado e transferem para os cuidadores responsabilidades que, em muitos casos, deveriam ser compartilhadas por redes institucionais mais estáveis e articuladas.

*A psicoterapia foi a mais difícil de conseguir. Pelo SUS, não havia profissionais especializados em autismo infantil disponíveis. Fui encaminhada para o CAPSij (Centro de Atenção Psicossocial Infantojuvenil), que está sobrecarregado e prioriza quadros psiquiátricos agudos. O Urano foi avaliado, mas por não se enquadrar nesse perfil de urgência clínica, acabou sendo ‘desregulado’ – ou seja, não incluído como prioridade. Diante disso, e vendo que ele apresentava sinais de ansiedade, agitação noturna e isolamento social, decidi buscar atendimento particular, com muito esforço. (...) A verdade é que o acesso às terapias pelo SUS existe, mas ele é cheio de obstáculos – fila de espera, descontinuidade dos atendimentos, falta de profissionais com especialização em autismo, e até dificuldade de deslocamento, já que nem todas as unidades são acessíveis ou próximas. É preciso insistência. É preciso saber o nome do setor certo, falar com o servidor certo, ter cópia de todos os laudos na bolsa o tempo todo. E mais do que tudo: é preciso energia. Energia que nem sempre temos.” (Gaia)*

*“Primeiro que a vida da gente é muito corrida e puxada. Tudo gira em torno do Eduardo. Ele exige cuidado o tempo todo. Não tem como sair e deixar ele com qualquer pessoa. Ele não fala, não anda, não dá pra largar com vizinho ou com neto, mesmo que o pessoal queira ajudar. A gente conhece cada sinal que ele dá, sabe o jeito dele. (...) A Helena vive com a saúde abalada, agora tá encostada, mas mesmo assim ajuda em tudo que pode. Eu, quando não tô com o Eduardo, tô tentando algum bico. Os filhos ajudam, mas cada um tem sua vida. Então a nossa vida é aqui dentro, com o menino, com os remédios, com os horários do posto, da APAE, das consultas.” (Atlas)*

Observa-se que, diante de falhas na coordenação dos serviços ou de mudanças nas condições de cuidado, as famílias são frequentemente levadas a reconfigurar suas estratégias, ajustando rotinas, acionando novas redes e redefinindo prioridades. Esses movimentos evidenciam que as condições intervenientes não atuam de forma estática, mas são continuamente reinterpretadas e enfrentadas no cotidiano, influenciando diretamente a forma como as normas de cuidado inclusivo são produzidas e sustentadas.

### O Tripé das Invenções Normativas em Ação (Estratégias de Ação/interação)

Os dados indicam que a invenção e a sustentação de normas de cuidado inclusivo se concretizam por meio de três estratégias de ação/interação interdependentes: coordenar rotinas (“planos vivos”), acionar redes e *advocacy* e performar a filiação. A partir das falas dos participantes, observa-se que essas estratégias não operam de forma isolada, mas se articulam continuamente no cotidiano, compondo um arranjo dinâmico que sustenta a organização do cuidado frente às demandas da criança e às condições do contexto.

*“Voltamos para nossa cidade, aquele cuidado todo de uma criança respiratória, a gente morava num bairro bom da cidade, então tinha uma boa qualidade de ar, as pessoas visitavam controladamente (...) fazia fisioterapia para recuperar o tempo internado, fazia em casa a fisioterapia (...). Quando voltamos, foi atrás de quem tinha, a gente conhecia o esposo dela, fizemos o contato, ela conseguiu a agenda para atender o Curetas.” (Orfeu)*

*A gente pediu uma semana para pensar. A gente precisa conversar entre a gente. Porque é uma mudança muito grande do que a gente tinha pensado. Então vamos sentar e conversar. Mas naquele momento foi definitivo quando a gente viu eles.” (Hermes)*

No que se refere à coordenação de rotinas, os dados mostram que as famílias constroem matrizes de organização do tempo e das atividades, articulando terapias, escola, deslocamentos, momentos de lazer e descanso. Observa-se que essa coordenação envolve antecipação de demandas, elaboração de alternativas diante de imprevistos e ajustes contínuos nas práticas de cuidado, contribuindo para a redução de rupturas e para maior previsibilidade no cotidiano.

*“Um exemplo muito claro aconteceu ontem, em uma atividade na escola do meu filho mais novo. Era uma festinha escolar: música alta, muita gente, bastante estímulo sensorial, um horário diferente do habitual, e a exigência de que as crianças fizessem apresentações para os pais. E entre as famílias presentes estava uma amiga nossa, que tem dois filhos pequenos, autistas, com diagnóstico recente. Durante a atividade, esses dois meninos entraram numa crise de desorganização. Estavam visivelmente angustiados, desorientados, reagindo ao excesso de estímulo que o ambiente impunha. Era barulho, era gente demais. E ali estava aquela mãe, completamente sozinha, tentando conter, acalmar, proteger os filhos sem apoio, sem acolhimento, sem estratégias. Aquilo me atravessou de um jeito profundo. Porque nós já vivemos isso com o Curetas. Já estivemos naquele lugar de desespero, de impotência, de solidão diante da crise do nosso filho.” (Réia)*

Observa-se, a partir das narrativas, que a responsividade institucional varia entre os diferentes serviços e momentos do percurso de cuidado, produzindo experiências que oscilam entre acolhimento e barreiras. Essa variação exige das famílias a construção de estratégias situadas para traduzir as necessidades da criança em linguagens reconhecidas pelos serviços, frequentemente mediadas por documentos como laudos, relatórios e planos institucionais.

*“A jornada para conseguir essas terapias começou no posto de saúde do nosso bairro. Levei os primeiros laudos (...) e solicitei os encaminhamentos pelo SUS. Foi aí que começou a maratona. (...) A terapia ocupacional também veio por encaminhamento do SUS, mas não na minha cidade. Consegui vaga em uma cidade vizinha, numa unidade conveniada, que atendeu Leandro por quase seis meses – até o contrato ser desfeito por questões burocráticas entre município e prestadora. Desde então, voltamos a depender da APAE (...) A psicoterapia foi a mais difícil de conseguir. Pelo SUS, não havia profissionais especializados em autismo infantil disponíveis. Fui encaminhada para o CAPSij (...), mas por não se enquadrar nesse perfil de urgência clínica, acabou sendo ‘desregulado’. (...) A verdade é que o acesso às terapias pelo SUS existe, mas ele é cheio de obstáculos – fila de espera, descontinuidade dos atendimentos, falta de profissionais com especialização em autismo e até dificuldade de deslocamento.” (Gaia)*

No que diz respeito ao acionamento de redes e *advocacy*, os dados indicam que as famílias mobilizam recursos formais e informais para viabilizar o cuidado, envolvendo parentes, profissionais, instituições e coletivos. Essa mobilização inclui tanto o estabelecimento de relações de apoio quanto a produção e circulação de documentos necessários para o acesso a serviços e direitos.

Os participantes evidenciam que a construção e a manutenção dessas redes exigem investimento contínuo, sendo fundamentais para ampliar o alcance das estratégias familiares e reduzir barreiras institucionais. Observa-se que a presença de atores-chave – como profissionais que facilitam o acesso a serviços – pode favorecer a continuidade do cuidado.

No que se refere à performação da filiação, os dados mostram que as famílias constroem, no cotidiano, práticas que afirmam o pertencimento da criança para além do vínculo jurídico formal. Essa estratégia é expressa em interações, narrativas e decisões que posicionam a criança como sujeito de direitos, sustentando sua inserção nos diferentes contextos de cuidado.

*“Faço uns trabalhos como pedreiro, ajudante, o que aparecer, porque a aposentadoria não dá para tudo. [...]. Então a semana gira muito em torno disso: comida, remédio, consulta, bico, e cuidar do Héspero.” (Atlas)*

Observa-se que essas práticas não apenas organizam o cuidado, mas também produzem reconhecimento e estabilidade simbólica, influenciando decisões relacionadas à priorização de atividades, ao manejo de demandas institucionais e à sustentação do vínculo familiar.

Os dados indicam que essas três estratégias – coordenação de rotinas, acionamento de redes e performação da filiação – operam de forma integrada, sendo continuamente ajustadas em resposta às condições intervenientes e aos eventos do cotidiano. Essa articulação constitui o núcleo operativo do modelo teórico, explicando como as famílias inventam e estabilizam normas de cuidado inclusivo em contextos marcados por variabilidade e incerteza*.*

### Quando as Normas se Materializam: Transformações Observáveis (Consequências)

Os dados indicam que, quando as estratégias de coordenação de rotinas, acionamento de redes e performação da filiação se articulam de forma mais estável, produzem transformações observáveis no cuidado, expressas em quatro dimensões inter-relacionadas: participação, previsibilidade, conforto e continuidade. A partir das falas dos participantes, observa-se que essas dimensões não operam de forma isolada, sendo construídas progressivamente no cotidiano das famílias.

*“Essa experiência, mais do que nos tornar pais de uma criança com deficiência, nos transformou como pessoas. Nos fez repensar valores, revisar preconceitos, e olhar a infância e a diversidade humana com muito mais profundidade e humanidade.” (Ártemis)*

No que se refere à participação, os dados mostram maior presença e engajamento da criança em diferentes contextos, como escola, atividades de lazer e convivência social, ainda que em níveis variados. Observa-se que essa ampliação está associada à capacidade das famílias de ajustar rotinas e negociar condições de inclusão nos serviços.

A previsibilidade, por sua vez, manifesta-se na redução de incertezas relacionadas a horários, fluxos de atendimento e organização das atividades, sendo construída por meio da antecipação de demandas e da consolidação de rotinas mais estáveis.

*“Começamos a ter momentos em família que incluíam todos – jogos, passeios, viagens curtas -, e isso foi aproximando os laços. O dia em que vi o Tritão ensinando o Jápeto a encaixar peças de um brinquedo educativo foi simbólico pra mim. Sem que ninguém pedisse, sem que houvesse uma câmera filmando ou uma plateia. Foi puro, natural, e cheio de afeto. Hoje, não tenho dúvidas: eles são irmãos. Não no papel apenas, mas no vínculo, no gesto, no olhar.” (Cronos)*

No que se refere ao conforto, os participantes indicam a redução de situações de sobrecarga sensorial e emocional, tanto para a criança quanto para a família, associada a adaptações no ambiente, na comunicação e na organização do cuidado. Já a continuidade do cuidado é observada na articulação mais consistente entre diferentes serviços e contextos, permitindo trajetórias menos fragmentadas ao longo do tempo.

*“Hoje, nossa rede de apoio é bem definida, embora não seja extensa. Contamos com poucas, mas confiáveis pessoas ao nosso redor – e isso tem sido suficiente [...].” (Atena)*

Observa-se, a partir das narrativas, que essas transformações não eliminam as dificuldades enfrentadas pelas famílias, mas indicam a construção de maior estabilidade no cuidado, ainda que provisória e sujeita a reconfigurações.

*“No começo, quando ele veio pra nossa casa e a gente assumiu a guarda, a gente nem sabia que ele tinha direito a essas terapias. Foi a assistente social lá do fórum que explicou pra gente. (...) O médico pediu encaminhamento pro neuro, pra fono, pra físio. Disseram que podia demorar um pouco – e demorou mesmo. Pra conseguir essas terapias, foi um caminho cheio de papelada. Tinha que levar laudo, comprovante de residência, número do benefício, xerox disso, xerox daquilo. Teve dia que a gente foi três vezes no mesmo lugar pra conseguir completar tudo.” (Atlas)*

Observa-se, a partir das narrativas, que a responsividade institucional varia entre os diferentes serviços e momentos do percurso de cuidado, produzindo experiências que oscilam entre acolhimento e barreiras. Essa variação exige das famílias a construção de estratégias situadas para traduzir as necessidades da criança em linguagens reconhecidas pelos serviços, frequentemente mediadas por documentos como laudos, relatórios e planos institucionais.

Os dados indicam também a presença de efeitos associados a esse processo, como a intensificação do trabalho de coordenação do cuidado e a necessidade contínua de lidar com demandas burocráticas e institucionais. Ao mesmo tempo, observa-se que a mobilização de redes e a sustentação do vínculo familiar podem contribuir para a redistribuição de responsabilidades e para a ampliação do suporte disponível.

*“A relação entre nossos filhos é, sem dúvida, uma das maiores provas de que o amor não depende da biologia [...]. Não foi apenas que “aceitaram” os irmãos. Eles se abriram de verdade” (Cronos)*

Por fim, os dados mostram que a estabilidade dessas transformações varia conforme as condições intervenientes e os contextos institucionais, sendo constantemente tensionada por eventos do cotidiano, que demandam novos ajustes e reconfigurações nas normas construídas pelas famílias.

## DISCUSSÃO

Este estudo propõe uma teoria substantiva que explica como e por que famílias adotivas de crianças com deficiência inventam normas de cuidado inclusivo para sustentar a vida cotidiana. Os resultados indicam que esse processo se organiza a partir da articulação de três estratégias de ação/interação – coordenação de rotinas (“planos vivos”), acionamento de redes e *advocacy* e performação da filiação – orientadas por uma ética do cuidado e moduladas por condições intervenientes presentes nos contextos institucionais e sociais.

Observou-se que essas estratégias não operam de forma isolada, mas constituem um arranjo dinâmico que permite às famílias responder às demandas da criança em cenários marcados por fragmentação dos serviços e descontinuidades no cuidado, aspecto amplamente descrito na literatura sobre cuidado a crianças com condições complexas e suas famílias^(18–20)^. Nesse sentido, os achados deslocam o foco de uma análise centrada apenas em barreiras ou impactos, evidenciando os mecanismos processuais pelos quais as famílias produzem participação, previsibilidade, conforto e continuidade ao longo do tempo^(21,22)^.

A partir dos resultados, compreende-se que as normas de cuidado inclusivo não se configuram como protocolos fixos, mas como arranjos situados, construídos na interação entre necessidades singulares da criança e exigências institucionais, em consonância com abordagens que destacam a centralidade da coordenação do cuidado e das experiências familiares nos sistemas de saúde^(21,22)^. Essa dinâmica pode ser interpretada à luz do Interacionismo Simbólico, ao evidenciar processos de definição de situações, negociação de papéis e construção de ação conjunta no cotidiano do cuidado^(20)^. Em diálogo, a perspectiva de Canguilhem contribui para compreender essas normas como expressões da capacidade de instituir novas referências de funcionamento diante de variações do “normal”^(14)^.

No campo da saúde, especialmente na Atenção Primária, esses achados reforçam o papel estratégico da Enfermagem na mediação entre famílias e serviços, na coordenação do cuidado e na construção de respostas mais sensíveis às necessidades concretas das crianças e de seus cuidadores, em alinhamento com evidências que apontam para a importância de práticas integradas e centradas na família^(4,22)^.

Ao conceber as normas de cuidado inclusivo como artefatos interacionais, os resultados evidenciam o trabalho contínuo de tradução, negociação e ajuste realizado pelas famílias para articular diferentes cenários de cuidado, como domicílio, escola, saúde e assistência social. Observou-se que esse processo envolve não apenas a organização prática das rotinas, mas também a mediação entre expectativas institucionais e necessidades singulares da criança, exigindo das famílias uma atuação ativa na coordenação do cuidado.

Os achados indicam que esse trabalho se intensifica em contextos marcados por fragmentação dos serviços e ausência de fluxos integrados, nos quais as famílias assumem a função de conectar pontos da rede e garantir a continuidade do cuidado, aspecto já descrito na literatura sobre encargos administrativos e experiências de usuários nos sistemas de saúde^(21)^. Nesse sentido, práticas como visitas domiciliares qualificadas, elaboração de Projetos Terapêuticos Singulares e acompanhamento longitudinal, embora reconhecidas como centrais, ainda se mostram subutilizadas na Atenção Primária, limitando a efetividade das ações e a possibilidade de construção de respostas mais ajustadas ao contexto familiar^(22,23)^.

A partir dos resultados, observa-se que as normas construídas pelas famílias operam como dispositivos práticos de estabilização do cuidado, permitindo alinhar rotinas, reduzir incertezas e sustentar a continuidade das ações ao longo do tempo. Esse movimento reforça evidências que destacam a centralidade da coordenação do cuidado e da participação familiar na produção de melhores desfechos em saúde, especialmente em contextos de maior complexidade^(4,21,22)^.

Os resultados explicitam um tripé operativo – coordenação de rotinas (“planos vivos”), acionamento de redes e *advocacy* e performação da filiação – como mecanismo central para a sustentação do cuidado ao longo do tempo. Observou-se que essas estratégias se articulam no cotidiano das famílias, permitindo lidar com múltiplas demandas simultâneas e responder a contextos institucionais frequentemente fragmentados, em consonância com evidências que apontam a centralidade da coordenação do cuidado e das intervenções centradas na família^(4,7,20,24)^.

A partir dos achados, identifica-se que a coordenação de rotinas funciona como um dispositivo organizador do cuidado, reduzindo rupturas e ampliando a previsibilidade das ações, enquanto o acionamento de redes e *advocacy* amplia o acesso a recursos e serviços, contribuindo para a continuidade do cuidado. Esses resultados dialogam com estudos que mostram que famílias de crianças com condições complexas frequentemente assumem a responsabilidade pela gestão do cuidado, mediando o acesso e articulando diferentes serviços ao longo do percurso assistencial^(22)^.

No que se refere à performação da filiação, os dados indicam que a construção cotidiana do pertencimento exerce papel central na sustentação do cuidado, influenciando decisões relacionadas à priorização de atividades, ao manejo das demandas institucionais e à organização das rotinas familiares. Esse aspecto amplia a compreensão do cuidado para além de sua dimensão técnica, aproximando-se de abordagens que destacam o papel das relações familiares, do vínculo e da comunicação na produção de cuidado em saúde^(4,24)^.

Observou-se ainda que a articulação entre essas três estratégias é orientada por uma ética do cuidado que atua como referência para a tomada de decisões em situações de conflito entre demandas institucionais e necessidades da criança, como já apontado em estudos que abordam o cuidado centrado na família e a necessidade de ajustes contextuais nas práticas assistenciais^(4,19)^. Nesse sentido, o tripé operativo identificado neste estudo contribui para explicar como as famílias constroem respostas situadas capazes de sustentar participação, previsibilidade, conforto e continuidade em contextos de elevada complexidade.

Do ponto de vista interacionista, os resultados evidenciam que a invenção de normas de cuidado inclusivo se constrói a partir de processos contínuos de definição de situações, negociação de papéis e construção de ação conjunta entre famílias, crianças e profissionais. Observou-se que, no cotidiano do cuidado, as famílias interpretam demandas institucionais, ajustam expectativas e redefinem responsabilidades, produzindo arranjos que permitem sustentar a organização do cuidado em contextos marcados por variabilidade e incerteza.

A partir dos achados, identifica-se que essas interações são mediadas tanto por experiências concretas – como o acesso a serviços e a responsividade institucional – quanto pela capacidade das famílias de traduzir suas necessidades em linguagens reconhecidas pelos sistemas de saúde, educação e assistência social. Esse movimento influencia diretamente quem tem legitimidade para participar das decisões e como o cuidado é organizado ao longo do tempo, em consonância com estudos que destacam a dimensão relacional e interacional da coordenação do cuidado^(20)^.

Observa-se ainda que a repetição de experiências – sejam elas de sucesso ou de dificuldade – contribui para a estabilização de determinadas práticas, permitindo às famílias consolidar rotinas, ajustar estratégias e sustentar normas construídas no cotidiano. Nesse sentido, os resultados indicam que a previsibilidade do cuidado não decorre apenas de protocolos institucionais, mas da capacidade de construir e manter acordos situados nas interações entre os diferentes atores envolvidos.

A partir dos resultados, a filosofia de Canguilhem contribui para compreender as normas de cuidado inclusivo como expressões da capacidade de instituir novas referências de funcionamento diante de variações do “normal”^(14)^. Observou-se que, quando as respostas institucionais não são suficientes para acomodar as necessidades da criança, as famílias passam a construir arranjos próprios – ajustando rotinas, reorganizando o ambiente e redefinindo prioridades – para sustentar o cuidado no cotidiano.

Nesse sentido, os achados indicam que tais normas não configuram desvios em relação aos padrões institucionais, mas formas legítimas de organização da vida, produzidas na interface entre condições concretas e possibilidades de ação das famílias. Essa perspectiva permite compreender que a continuidade do cuidado não depende apenas da adesão a protocolos, mas da capacidade de criar soluções situadas que respondam às demandas específicas de cada contexto, em consonância com a noção de normatividade do viver proposta por Canguilhem^(14)^.

Observa-se ainda que esses processos de invenção normativa intensificam-se em cenários marcados por descontinuidades nos serviços e exigências institucionais pouco flexíveis, nos quais as famílias assumem um papel ativo na construção de alternativas para sustentar o cuidado^(25,26)^. Dessa forma, os resultados reforçam a importância de reconhecer essas práticas não apenas como estratégias adaptativas, mas como produções normativas que podem orientar respostas mais sensíveis e contextualizadas nos serviços de saúde.

Este estudo apresenta limitações que devem ser consideradas na interpretação dos achados. Trata-se de uma investigação qualitativa, realizada com um número restrito de participantes e em um único município, o que não permite generalizações, embora favoreça a compreensão aprofundada do fenômeno em seu contexto^(27–30)^. Além disso, a análise foi construída a partir da perspectiva de cuidadores, não incluindo diretamente a voz das crianças e adolescentes, o que pode limitar a apreensão de dimensões importantes do cuidado. Ainda assim, a utilização da *Grounded Theory* e o alcance de saturação teórica conferem robustez analítica aos resultados, possibilitando a construção de um modelo explicativo sensível às variações e condições do fenômeno^(9–11)^.

Os resultados deste estudo indicam que a invenção de normas de cuidado inclusivo constitui um processo relacional, dinâmico e situado, sustentado pela articulação entre estratégias familiares, condições intervenientes e contextos institucionais. Ao explicitar os mecanismos processuais que organizam o cuidado no cotidiano, o modelo proposto contribui para compreender como se produzem participação, previsibilidade, conforto e continuidade em contextos marcados por fragmentação dos serviços e exigências institucionais.

## CONSIDERAÇÕES FINAIS

Este estudo contribui para a compreensão de como e por que famílias adotivas de crianças com deficiência inventam e estabilizam normas de cuidado inclusivo no cotidiano. Ao explicitar o papel da coordenação de rotinas, da mobilização de redes e da construção da filiação na sustentação do cuidado, o modelo proposto amplia a análise dos mecanismos que produzem participação, previsibilidade, conforto e continuidade em contextos marcados por fragmentação institucional.

No campo da Enfermagem, os achados redirecionam o papel do enfermeiro na Atenção Primária à Saúde para a função de Gestor de Casos (Case Manager) e Navegador em Saúde. A atuação deste profissional deve transcender o cuidado clínico direto, centrando-se na coordenação proativa de fluxos intersetoriais e na mediação técnica entre as famílias e a rede de serviços (saúde, educação e assistência social). A Enfermagem de Família, ao identificar precocemente lacunas assistenciais e sobrecarga dos cuidadores, tem o potencial de reduzir a necessidade de as famílias “inventarem” normas isoladamente, promovendo a estabilização do cuidado por meio de planos terapêuticos integrados que garantam a previsibilidade e a continuidade assistencial.

Recomenda-se o desenvolvimento de estudos longitudinais, comparativos e de implementação, bem como investigações que incorporem a perspectiva de crianças e adolescentes, a fim de aprofundar a compreensão dos processos de cuidado e das condições que favorecem sua sustentabilidade.

## Data Availability

Todo o conjunto de dados que dá suporte aos resultados deste estudo está disponível mediante solicitação ao autor correspondente.

# Translation: Creación de normas de atención inclusiva en la adopción de niños con discapacidad: coordinación de rutinas, redes y filiación*

**DOI:** 10.1590/1980-220X-REEUSP-2025-0554es

**Authors:** Luciano Silveira Pacheco de Medeiros, Giovana Calcagno Gomes, Max dos Santos Afonso, Thiago Ferreira Abreu, Jacqueline Flores de Oliveira, Francisco Rosemiro Guimarães Ximenes, Luciano Garcia Lourenção, Clarice Alves Bonow

**Affiliations:** 1 Universidade Federal de Pelotas, Faculdade de Enfermagem, Programa de Pós-Graduação em Enfermagem, Pelotas, RS, Brazil.; 2 Universidade Federal do Rio Grande, Escola de Enfermagem, Programa de Pós-Graduação em Enfermagem, Rio Grande, RS, Brazil.; 3 Universidade Católica de Pelotas, Faculdade de Medicina, Pelotas, RS, Brazil.; 4 Universidade Estadual Vale do Acaraú, Centro de Ciências da Saúde. Sobral, CE, Brazil.

**Keywords:** Adopción, Niños con Discapacidad, Atención Primaria de Salud, Cuidados en el Hogar de Adopción, Enfermería

## Abstract

**Objetivo:**

Analizar cómo y por qué las familias adoptivas de niños con discapacidad inventan y establecen normas de atención inclusiva en su vida diaria.

**Método:**

Un estudio cualitativo, basado en la Teoría Fundamentada, realizado en un municipio del sur de Brasil, con 11 familias adoptivas. Se realizaron entrevistas individuales, presenciales y semiestructuradas, que se analizaron mediante comparación constante y codificación abierta, axial y selectiva.

**Resultados:**

El análisis reveló una teoría sustantiva que explica la invención de normas de atención inclusivas como un proceso relacional, dinámico y situado. El modelo vincula las causas, el contexto y las condiciones intervinientes con estrategias de acción/interacción guiadas por una ética del cuidado, expresada en tres movimientos: coordinar rutinas, activar redes y *advocacy*, y establecer vínculos de filiación. Estos movimientos generan participación, previsibilidad, comodidad y continuidad, lo que permite mantener la atención en contextos marcados por la fragmentación institucional.

**Conclusión:**

La invención de normas de atención inclusiva expresa la producción de respuestas contextualizadas a las necesidades del niño y a las limitaciones institucionales. Los resultados refuerzan la importancia de la coordinación de la atención, la participación familiar y el papel de la enfermería como mediadora entre las familias y los servicios.

## INTRODUCCIÓN

La adopción de niños con discapacidad constituye un ámbito privilegiado para comprender cómo se negocian y recrean las normas sociales, institucionales y familiares en el día a día de la atención. Al entrecruzar la filiación adoptiva y la discapacidad, surgen capas jurídicas, morales, clínicas, escolares y asistenciales que definen quién cuida, en qué condiciones y con qué reconocimiento^(1)^. En redes heterogéneas y asimétricas, las familias suelen verse llamadas a articular exigencias de previsibilidad y continuidad con prácticas que aseguren la participación y el bienestar del niño^(2–4)^.

En este contexto, la familia puede entenderse como un sistema relacional en constante negociación, constituido por vínculos de filiación, cuidado y pertenencia, cuya organización se da en el entrelazamiento entre las experiencias cotidianas y las exigencias institucionales^(3)^. Al asumir la coordinación del cuidado en entornos a menudo fragmentados, las familias ponen en marcha estrategias que sustentan el compromiso, la participación y la continuidad de las acciones, en consonancia con los principios del cuidado centrado en la familia^(2,4)^.

Los datos del Sistema Nacional de Adopción y Acogimiento (SNA) de 2025/2026 muestran que Brasil cuenta con cerca de 34 000 solicitantes habilitados para aproximadamente 5 000 niños aptos para la adopción^(5)^. Sin embargo, el perfil de salud sigue siendo el principal obstáculo. Se estima que alrededor del 25 % de los niños y adolescentes en la lista de espera para la adopción tienen alguna discapacidad o enfermedad crónica, mientras que menos del 5 % de los solicitantes aceptan este perfil^(6)^. Este desajuste cuantitativo pone de manifiesto la vulnerabilidad de estos niños y adolescentes, quienes enfrentan tiempos de espera más prolongados y exigen a las familias adoptivas una profunda reorganización normativa para subsanar las brechas en la atención.

Aunque este escenario pone de manifiesto el papel central de las familias en el sustento del cuidado, los procesos mediante los cuales estas producen, negocian y estabilizan las respuestas cotidianas siguen sin comprenderse lo suficiente. A pesar de los avances en la literatura sobre la descripción de los impactos, las barreras y las desigualdades asociadas a la discapacidad, aún son escasas las explicaciones sobre los mecanismos relacionales y prácticos que permiten a las familias construir, organizar y sostener el cuidado en contextos marcados por apoyos institucionales parciales, intermitentes o descoordinados^(7,8)^.

En este estudio, se entienden las normas de cuidado inclusivo como arreglos prácticos y simbólicos que orientan las decisiones y rutinas de la vida cotidiana, generados en la interfaz entre las necesidades singulares del niño y los patrones institucionales. Estas normas se expresan, de manera articulada, en la organización de las rutinas, en la movilización de redes y en la construcción cotidiana de la filiación, contribuyendo a generar participación, previsibilidad, comodidad y continuidad en el cuidado^(2,4)^.

Ante este panorama, la pregunta que orienta este estudio es: ¿cómo y por qué las familias adoptivas de niños con discapacidad inventan y consolidan normas de cuidado inclusivo en la vida cotidiana? Así, el objetivo es analizar cómo y por qué las familias adoptivas de niños con discapacidad inventan y consolidan normas de cuidado inclusivo.

## MÉTODO

### Diseño del Estudio

Estudio cualitativo, basado en la *Teoría fundamentada* (Grounded Theory), en su vertiente straussiana, orientado a la construcción de una teoría sustantiva sobre el fenómeno de la tesis “*Inventando normas de cuidado inclusivo— coordinando rutinas, activando redes y representando la filiación—para aumentar la participación, la previsibilidad, la comodidad y la continuidad*”^(9:página)^. La elección de la *Teoría fundamentada* se justifica por la necesidad de explicar procesos relacionales, dinámicos y situados en contextos de fragmentación institucional, lo que permite que el modelo teórico articule condiciones, estrategias y consecuencias del cuidado^(9)^.

El diseño, centrado en procesos sociales e interaccionales, permite captar la variación, las condiciones y las consecuencias en cadena mediante la comparación constante, el muestreo teórico y los ciclos de codificación abierta, axial y selectiva^(9,10)^, con la elaboración de notas y la búsqueda de la saturación teórica^(11)^.

En el plano teórico-interpretativo, se utilizan conceptos sensibilizadores—y no categorías a priori para la codificación—procedentes del IS y de la obra de Georges Canguilhem, con el fin de profundizar en la explicación de los hallazgos sin dirigir la generación de los códigos^(12–14)^.

### Población del Estudio

El estudio contó con 11 participantes (seis mujeres y cinco hombres), con predominio de profesionales de la educación (profesoras y profesores universitarios, una maestra de la red pública y una pedagoga), seguidos de profesionales de la salud (dos médicos), además de un albañil jubilado y una ama de casa. Las estructuras familiares observadas fueron: adopciones por parejas heterosexuales (nueve casos), adopción por una pareja homosexual (un caso), adopción por una madre soltera (un caso) y adopción intrafamiliar intergeneracional (un caso). En total, hubo 16 niños adoptados—contando los hermanos compartidos por parejas entrevistadas solo una vez—. En cuanto a las condiciones, prevalecen los casos de parálisis cerebral (PC) con co-ocurrencia de trastorno del espectro autista (TEA)—niveles I a III—y discapacidad intelectual, con co-ocurrencia de trastorno por déficit de atención e hiperactividad (TDAH), también en co-ocurrencia con TEA.

### Lugar del Estudio

El estudio se llevó a cabo en un municipio del sur de Brasil. Las trayectorias de atención de las familias participantes se articulan, en el propio municipio, a través de interfaces intersectoriales que incluyen salud (Atención Primaria de Salud—APS—, servicios ambulatorios especializados, centros de rehabilitación y hospitales pediátricos), educación (escuelas públicas y privadas, atención educativa especializada), asistencia social (Centro de Referencia de Asistencia Social—CRAS—, Centro de Referencia Especializado de Asistencia Social—CREAS—, prestaciones y servicios de protección social) y el sistema de garantía de derechos (Consejo Tutelar, Ministerio Público y Juzgado de la Infancia y la Juventud).

Este marco es fundamental para la participación y la continuidad del cuidado del niño, pero a menudo se caracteriza por la fragmentación organizativa y las discontinuidades, lo que exige un trabajo adicional de coordinación por parte de las familias y repercute en su calidad de vida y su participación comunitaria.

### Criterios de Selección

Se incluyeron a los cuidadores principales de familias adoptivas de niños con discapacidad—congénita o adquirida, transitoria o permanente, física, intelectual, físico-intelectual o múltiple—cuya condición pudiera ser anterior a la adopción o haberse observado o diagnosticado durante la etapa de convivencia. Se excluyeron las familias en proceso de adopción, en la etapa de convivencia, y las familias bajo tutela judicial.

Se inició el reclutamiento de participantes mediante la colaboración de instituciones de salud y educación que atienden a personas con discapacidad. Tras la presentación detallada del proyecto a los responsables institucionales, se solicitó su apoyo para identificar a las familias que cumplieran con los criterios de elegibilidad. Las indicaciones iniciales se complementaron mediante la técnica de muestreo en bola de nieve (*snowball*)^(15)^, en la que los participantes indicaban a nuevos participantes potenciales.

El muestreo teórico se llevó a cabo de manera progresiva y se orientó por las categorías emergentes a lo largo del análisis inicial. Las primeras entrevistas pusieron de manifiesto la importancia central del trabajo de coordinación cotidiana y de la mediación institucional en el cuidado, lo que condujo a la búsqueda deliberada de variación en los arreglos familiares y las condiciones contextuales capaces de poner a prueba y ampliar estas características. Así, se incluyeron configuraciones distintas— parejas heterosexuales, pareja homosexual, madre soltera y adopción intergeneracional—, así como diferentes combinaciones de discapacidades, niveles de dependencia y trayectorias institucionales, con el objetivo de explorar cómo se manifestaba el fenómeno en condiciones diversas. Las decisiones sobre la muestra se reevaluaron continuamente a la luz de los memorandos analíticos, que señalaban lagunas en la densidad teórica, la necesidad de contrastar casos o de profundizar en dimensiones aún poco exploradas, en consonancia con el principio de comparación constante característico de la *Teoría fundamentada straussiana* ^(9)^.

El contacto inicial con los candidatos seleccionados se realizó mediante mensajes de *WhatsApp* ^®^, en los que el investigador se presentó, detalló su trayectoria académica y profesional, y expuso las motivaciones—tanto académicas como personales—que orientaron la investigación. En este primer contacto, también se presentaron los objetivos y la finalidad del estudio, estableciendo una comunicación clara y transparente.

### Recolección de Datos

La recolección se llevó a cabo entre noviembre de 2023 y junio de 2024. Se utilizaron entrevistas presenciales, individuales, semiestructuradas y en profundidad, con 33 preguntas abiertas. Cuatro de las entrevistas se realizaron en los lugares de trabajo de los participantes y siete en sus hogares. De las entrevistas realizadas en los hogares, solo dos participantes estaban acompañados: Atlas, acompañado de su hijo adoptivo, y Prometeo, acompañado de su madre, una señora de 94 años. Se realizó una entrevista piloto con un participante y no se observó la necesidad de ajustes en el guion de preguntas. Dado que se trataba de una población de estudio bastante pequeña y de un tema delicado, este participante fue incluido en el grupo final de participantes. No hubo necesidad de repetir ninguna de las entrevistas.

El guion se elaboró a partir del objetivo del estudio y se perfeccionó mediante muestreo teórico tras lecturas preliminares, notas y comparaciones constantes (caso por caso y entre casos), manteniendo preguntas abiertas sobre: (a) la decisión de adoptar y la reconfiguración de proyectos; (b) los contextos sociales, institucionales y simbólicos del cuidado; (c) redes y *advocacy* e interacciones con los servicios; (d) coordinación de rutinas (“planes vivos”); y (e) efectos y transformaciones percibidas.

Las entrevistas fueron realizadas por un profesional de enfermería y doctorando en Enfermería, con experiencia y pericia en investigación cualitativa por parte del grupo de investigación participante, sin vínculo asistencial ni afectivo con los participantes. Las entrevistas tuvieron una duración promedio de 120 a 150 minutos. Se grabaron y transcribieron manualmente en formato semi-verbal, con doble verificación de calidad y anonimización mediante seudónimos. Inmediatamente después de cada contacto se elaboraron memorandos y notas—analíticas y descriptivas—. La recolección de datos concluyó por saturación teórica cuando las categorías centrales presentaron suficiente densidad en términos de propiedades, dimensiones y relaciones entre condiciones, estrategias y consecuencias. A partir de la novena entrevista, se observó que las nuevas narrativas tendían a reiterar variaciones de propiedades ya identificadas—especialmente en lo que se refiere a la coordinación de rutinas, la mediación institucional y la representación de la filiación—sin introducir categorías sustancialmente nuevas. Las dos entrevistas posteriores sirvieron como prueba de solidez del modelo en construcción, confirmando relaciones ya establecidas y ampliando los ejemplos empíricos, pero sin alterar su arquitectura conceptual. En ese momento, se consideró alcanzada la saturación analítica, entendida como la estabilidad teórica de las categorías centrales^(11)^. No hubo rechazos.

El proceso de validación de los datos no incluyó la devolución de las transcripciones completas a los participantes. En su lugar, la fiabilidad del contenido se aseguró mediante la discusión de las percepciones y observaciones del investigador con cada participante. Se analizaron, en conjunto, las fortalezas y debilidades observadas en el proceso de Recolección de datos.

### Análisis y Procesamiento de los Datos

El análisis y el procesamiento de los datos se realizaron manualmente. Se llevaron a cabo lecturas iterativas para segmentar en unidades de sentido, preservando los contextos del discurso, y se inició la codificación con un registro sistemático en memorandos que documentaban las decisiones analíticas y las hipótesis emergentes^(9,10)^.

La codificación abierta se realizó línea por línea, dando prioridad a los códigos *in vivo* y a la comparación constante dentro de las entrevistas y entre ellas para captar la variación, las condiciones y las consecuencias del fenómeno^(10)^. Los memorandos funcionaron como dispositivos de trazabilidad, orientando el muestreo teórico a lo largo de la Recolección. En la codificación axial, se articularon las propiedades y dimensiones de los códigos en categorías y subcategorías, utilizando el paradigma condicional/consecuencial para modelar relaciones y procesos a lo largo del tiempo^(9)^.

La codificación selectiva integró las categorías en torno al fenómeno central—”Inventar normas de cuidado inclusivo—coordinando rutinas, activando redes y manifestando filiación—para aumentar la participación, la previsibilidad, la comodidad y la continuidad”—, lo que dio como resultado el modelo teórico sustantivo^(9)^. En el plano teórico-interpretativo, se utilizaron conceptos del Interaccionismo Simbólico (IS) y de la filosofía de Georges Canguilhem como lentes analíticas para comprender los procesos de negociación, construcción de significados y producción normativa implicados en el cuidado^(13,14)^. El IS permitió acceder a los significados atribuidos a las interacciones cotidianas, mientras que la perspectiva de Canguilhem fundamentó el análisis de la “invención de normas” como una respuesta vital y normativa de las familias ante las adversidades del entorno.

A lo largo del proceso analítico, códigos in vivo como “hacer que la vida siga adelante”, “ocuparse del papeleo” y “organizar todo para que no se descarrile” se registraron inicialmente en la codificación abierta como acciones situadas de coordinación. En la etapa axial, dichos códigos se agruparon bajo la categoría “coordinar intensamente la vida cotidiana”, articulándose con condiciones intervinientes como la fragmentación institucional y las exigencias burocráticas. La comparación constante entre los casos reveló que esta coordinación no operaba de manera aislada, sino que se integraba a la movilización de redes y a la construcción de la filiación como estrategia de estabilización del cuidado. En la codificación selectiva, estas relaciones convergieron en la formulación del fenómeno central—”Inventando normas de cuidado inclusivo”—, entendido como el proceso mediante el cual las familias establecen acuerdos normativos locales para sustentar la participación, la previsibilidad, el confort y la continuidad. Este movimiento integró categorías, condiciones y consecuencias en un modelo teórico procesual.

La conducción analítica siguió la vertiente straussiana de la *Grounded Theory*, anclada en el pragmatismo y en el IS, dando prioridad a la emergencia conceptual a partir de los datos empíricos. Las categorías se construyeron mediante la comparación constante y el refinamiento progresivo, sin la aplicación previa de estructuras teóricas externas. Dichos marcos de referencia se utilizaron como conceptos sensibilizadores y no como categorías a priori para la codificación, con el fin de profundizar en la explicación de los hallazgos sin dirigir la generación de los códigos, preservando así la lógica inductiva y procesual que caracteriza el enfoque adoptado^(8,13,14)^.

La recolección de datos concluyó al alcanzar una densidad teórica suficiente para sustentar el análisis orientado por la pregunta central. El estudio siguió rigurosamente los estándares del COREQ: 32 ítems para estudios cualitativos basados en entrevistas^(16)^.

### Aspectos Éticos

Los procedimientos se ajustaron a la Resolución del Consejo Nacional de Salud (CNS) n.º 510/2016^(17)^, con la aprobación del Comité de Ética en Investigación de la Facultad de Enfermería de la Universidad Federal de Pelotas—Dictamen n.º 6.532.379; CAAE 75718623.8.000.5316. Se informó a todos y todas sobre los resultados parciales y finales de la investigación y de que estos se darían a conocer en medios técnico-científicos. El conjunto completo de datos que respalda los resultados de este estudio se publicó en el mismo artículo.

Para preservar la voz de los participantes y, al mismo tiempo, garantizar la desidentificación, los extractos presentados se editaron únicamente para eliminar marcas identificativas y realizar pequeños ajustes de legibilidad, sin alterar el sentido; los nombres propios se sustituyeron de acuerdo con el protocolo de anonimización adoptado.

## RESULTADOS

El análisis de las entrevistas reveló una teoría sustantiva que articula causas, contexto y condiciones intervinientes a nivel operativo de las estrategias de acción/interacción, orientadas por una ética del cuidado. Los hallazgos evidenciaron que las familias adoptivas de niños con discapacidad construyen y sostienen normas de cuidado inclusivo a través de tres movimientos interdependientes: coordinar rutinas (“planes vivos”), activar redes y *advocacy*, y poner en práctica la filiación. Estos movimientos, cuando se articulan, producen efectos observables de participación, previsibilidad, comodidad y continuidad del cuidado.

Los datos indican que este proceso no ocurre de manera lineal. Por el contrario, los eventos clave—como crisis clínicas, interrupciones escolares, cambios en el acceso a prestaciones y transiciones en el cuidado—desencadenan reconfiguraciones en las estrategias adoptadas por las familias, lo que exige ajustes continuos en las normas establecidas. Se observa que dichos eventos funcionan como puntos críticos de inflexión, reabriendo decisiones y generando tensiones en los arreglos previamente estabilizados.

En este contexto, las mediaciones organizacionales—relacionadas con el funcionamiento de los servicios de salud, educación y asistencia social—modulan el alcance de las estrategias familiares, pudiendo facilitar o dificultar la continuidad del cuidado. A partir de los testimonios de los participantes, se identifica que la capacidad de articulación entre diferentes puntos de la red influye directamente en la estabilidad de las normas construidas en la vida cotidiana.

Para facilitar la comprensión de este proceso, se presenta el modelo teórico en la Figura 1, que sintetiza las relaciones entre las dimensiones analíticas identificadas. En el modelo, las estrategias de acción/interacción—coordinar rutinas (“planes vivos”), activar redes y *advocacy*, y poner en práctica la filiación—se articulan a partir de causas, contexto y condiciones intervinientes, produciendo consecuencias observables: participación, previsibilidad, comodidad y continuidad del cuidado. Las flechas indican tanto el flujo principal como los movimientos de retroalimentación activados por eventos centinela.

**Figura 1:** – Modelo teórico del cuidado inclusivo en la adopción. Pelotas, RS, Brasil, 2025.

**Fuente:** Elaboración propia.

### Punto de Partida: Decisiones, Valores y Reorientaciones (Causas)

Los datos indican que la decisión de adoptar a un niño con discapacidad constituye un punto de inflexión en los proyectos de parentalidad, lo que genera procesos de reconfiguración de valores, expectativas y formas de vida. A partir de los testimonios de los participantes, se observa que esta decisión no se reduce a un evento puntual, sino que forma parte de trayectorias marcadas por reflexiones sobre justicia, cuidado y responsabilidad, que orientan la construcción de nuevas referencias para el ejercicio de la parentalidad.

*“La decisión de adoptar surgió durante el período en que vivíamos en Brasilia, debido a un proyecto profesional mío, que en ese momento requeriría pasar algunos años fuera [...]. Estábamos en una etapa de estabilidad emocional y profesional, y hablábamos con cierta frecuencia sobre la posibilidad de ampliar la familia. La idea de la adopción no surgió de un deseo repentino, ni de una motivación específica como la infertilidad o algo por el estilo”. (Atena)*

Se observa que, en este proceso, las familias comienzan a revisar las expectativas relacionadas con el desarrollo, la escolarización y la autonomía del niño, sustituyendo las metas previamente idealizadas por horizontes más flexibles y ajustados a las condiciones concretas del cuidado. Esta reconfiguración implica también cambios prácticos, como la reorganización de las rutinas laborales, adaptaciones en el espacio doméstico y la incorporación progresiva de conocimientos sobre la discapacidad, a menudo construidos a partir de la experiencia cotidiana.

*“La casa donde vivimos hoy nos la cedió mi mamá, [...] pero no estaba preparada para acoger a una niña con discapacidad. De hecho, creo que ninguna casa lo está. Vamos descubriendo lo que necesitamos a medida que se desarrolla la convivencia. Es como si el amor fuera revelando las necesidades, una por una. Antes de la llegada de nuestra hija, hicimos una reforma inicial. Como tiene movilidad reducida, aunque puede caminar distancias cortas con ayuda, sabíamos que tendríamos que adaptar su baño”. (Héstia)*

A partir de los relatos, se identifica que estas transformaciones no ocurren de manera estática, sino que se actualizan continuamente ante nuevas demandas diagnósticas, requisitos institucionales y experiencias vividas en el día a día del cuidado. Este proceso sustenta la construcción de disposiciones para la creación de normas locales, entendidas como arreglos prácticos que permiten a las familias responder a las necesidades de la niña en contextos a menudo marcados por lagunas y discontinuidades en los servicios.

### Escenas del Cuidado: Niveles Normativos y Escenarios de Práctica (Contexto)

Los datos indican que el cuidado de los niños adoptados con discapacidad se desarrolla en múltiples escenarios intersectoriales, que abarcan el hogar, la escuela, los servicios de salud, la asistencia social y el sistema de justicia. A partir de los testimonios de los participantes, se observa que estos espacios no funcionan de manera aislada, sino que constituyen un campo relacional en el que las normas formales, las rutinas institucionales y las prácticas cotidianas se superponen, coexisten y, en ocasiones, entran en conflicto.

En este contexto, los protocolos escolares, los flujos de la red de salud, los dispositivos de asistencia social y las normativas legales relacionadas con la adopción pasan a organizar el acceso, los tiempos y las responsabilidades en el cuidado. Sin embargo, los datos evidencian que dichas normativas no son del todo estables ni suficientes, lo que exige a las familias un trabajo continuo de mediación, negociación y adaptación para hacer posible la participación y la continuidad del cuidado.

*“Al principio, la escuela parecía acogedora. Usaba todas las palabras correctas: inclusión, diversidad, afecto, escucha. Pero en la práctica las cosas resultaron mucho más difíciles. Urano, a los ojos de los maestros, era ‘difícil’. No seguía las instrucciones con facilidad, tenía crisis cuando se alteraba la rutina, no interactuaba de la forma esperada con los otros niños. Y, como no hablaba, muchas cosas se interpretaban como desobediencia, como falta de atención, como berrinches. A partir de ahí, entendí que mi relación con la escuela no podía ser pasiva”. (Gaia)*

A partir de los relatos, se observa que la capacidad de respuesta institucional varía entre los diferentes servicios y momentos del proceso de atención, lo que genera experiencias que oscilan entre la acogida y las barreras. Esta variación exige que las familias desarrollen estrategias específicas para traducir las necesidades del niño a un lenguaje reconocido por los servicios, a menudo mediado por documentos como dictámenes, informes y planes institucionales.

*“Sí, después de adoptar a Hécate, empezamos a necesitar varios otros tipos de servicios además de los de salud, principalmente en las áreas de educación y asistencia social. Su llegada no solo cambió nuestro hogar, sino también la forma en que nos relacionamos con la red pública de apoyo. Acudimos a la Secretaría Municipal de Educación para entender cómo funcionaría su inscripción en la escuela. Como tiene parálisis cerebral y limitaciones motoras y cognitivas, sabíamos que tendría derecho a la educación inclusiva, pero no sabíamos cómo se llevaría a cabo en la práctica”. (Deméter)*

*“Para empezar, tan pronto como quedó bajo nuestra custodia, el personal del juzgado ya nos dijo que buscáramos el CRAS. Fue allí donde nos ayudaron con el proceso para obtener el BPC* [Beneficio de Prestación Continua]*, que es ese beneficio para personas con discapacidad. Fueron trámites por aquí, informes por allá, visitas de la trabajadora social a casa. Ese dinero es hoy lo que nos permite mantener muchas cosas aquí. Sin él, no podríamos cubrir lo básico. El CRAS también nos ayudó con una canasta básica, sobre todo al principio, cuando nos quedamos con el niño sin tener ni pañales que darle. Vinieron aquí, vieron nuestra situación y trajeron ayuda. No fue mucho, pero en ese momento nos salvó”. (Atlas)*

Los datos muestran que, en este ámbito institucional-familiar, las prácticas de cuidado en el hogar—como la organización del espacio, el uso de tecnologías de apoyo y la creación de rutinas—se articulan continuamente con exigencias externas. Cuando existe un desajuste entre estas esferas, surgen tensiones que exigen ajustes y reconfiguraciones en las formas de cuidado, lo que pone de manifiesto el carácter dinámico y negociado del contexto en el que se producen las normas de cuidado inclusivo.

### Fuerzas que Modulan el Alcance (Condiciones Intervinientes)

Los datos indican que la capacidad de las familias para establecer y mantener normas de cuidado inclusivo no depende únicamente de sus decisiones o estrategias, sino que está fuertemente modulada por un conjunto de condiciones intervinientes que amplían o restringen el alcance del cuidado en la vida cotidiana. A partir de los testimonios de los participantes, se observa que dichas condiciones se expresan en dimensiones materiales, relacionales, institucionales y simbólicas, y operan de manera combinada a lo largo de las trayectorias de cuidado.

Entre estas condiciones, algunas actúan como facilitadoras, ampliando el acceso a los recursos, fortaleciendo las redes de apoyo y favoreciendo la continuidad del cuidado. Otras operan como barreras importantes, manifestándose en la insuficiencia de servicios, la fragmentación de las redes de asistencia, las dificultades de acceso, la sobrecarga familiar y la discontinuidad de los seguimientos. Los datos indican que la forma en que se combinan estas condiciones influye directamente en la capacidad de las familias para mantener normas de cuidado inclusivo a lo largo del tiempo.

*“Sí, actualmente nuestros hijos reciben terapias, sobre todo Orión, quien tiene un diagnóstico de autismo y requiere una atención más intensiva en diversas áreas del desarrollo. Desde el principio, nos dimos cuenta de la importancia de garantizarle un seguimiento multidisciplinario y especializado, capaz de ofrecerle los estímulos y el apoyo que necesita para desarrollarse con mayor autonomía y calidad de vida. Órion recibe diversas terapias: seguimiento con un neuropediatra, una fonoaudióloga, un terapeuta ocupacional y una psicóloga. Cada profesional tiene un papel importante y, juntos, forman una red de apoyo técnico que, poco a poco, ha contribuido a avances significativos en su vida”. (Ártemis)*

A partir de los relatos, se observa que la coordinación entre profesionales y servicios constituye un elemento central en la modulación de la atención, pudiendo favorecer la construcción de rutinas más estables o, por el contrario, generar discontinuidades cuando no existe o se debilita.

*“En realidad, dejó las sesiones terapéuticas poco después de que le dieran de alta del CAPS, poco tiempo después de que lo adoptáramos. Durante el período en que estuvo bajo un seguimiento más intenso, tuvo acceso a diferentes tipos de terapias, tanto en el CAPS como en el CER (Centro Especializado de Rehabilitación). En el CER y en el CAPS, recibió varios tipos de atención. Tuvo sesiones regulares de fonoaudiología durante un tiempo. Era evidente cuánto le ayudaba eso a sentirse más seguro al hablar, más comprendido por las personas que lo rodeaban. Además, también recibió acompañamiento psicológico.” (Prometeu)*

Los datos indican además que las experiencias de acogida o estigma en los servicios influyen en el protagonismo de las familias, afectando su disposición para reclamar derechos, negociar con las instituciones y mantener estrategias de cuidado a lo largo del tiempo. En este sentido, el acceso a la información, el dominio de los trámites burocráticos y la capacidad de movilizar recursos institucionales se configuran como elementos que diferencian las trayectorias más estables de aquellas marcadas por un mayor desgaste y discontinuidad.

Aunque parte de los participantes relató experiencias positivas con servicios especializados y redes de apoyo, los datos también evidencian un conjunto de condiciones intervinientes que dificultan y limitan la continuidad y la efectividad del cuidado. Entre ellas se destacan la falta de profesionales especializados, la sobrecarga de los servicios públicos, la fragmentación de los flujos de atención, las interrupciones terapéuticas, las dificultades de desplazamiento y la necesidad constante de mediación burocrática por parte de las familias. Estas condiciones generan un desgaste acumulado y transfieren a los cuidadores responsabilidades que, en muchos casos, deberían ser compartidas por redes institucionales más estables y coordinadas.

*La psicoterapia fue lo más difícil de conseguir. A través del SUS, no había profesionales especializados en autismo infantil disponibles. Me derivaron al CAPSij (Centro de Atención Psicosocial para Niños y Adolescentes), que está sobrecargado y da prioridad a los casos psiquiátricos agudos. A Urano lo evaluaron, pero al no encajar en ese perfil de urgencia clínica, terminó siendo “desclasificado”—es decir, no se lo incluyó como prioridad—. Ante esto, y al ver que presentaba signos de ansiedad, agitación nocturna y aislamiento social, decidí buscar atención privada, con mucho esfuerzo. (...) La verdad es que el acceso a las terapias a través del SUS existe, pero está lleno de obstáculos: listas de espera, discontinuidad en la atención, falta de profesionales especializados en autismo e incluso dificultades de desplazamiento, ya que no todas las unidades son accesibles o están cerca. Se necesita insistencia. Hay que saber el nombre del departamento correcto, hablar con el funcionario adecuado, tener una copia de todos los informes en la cartera en todo momento. Y, sobre todo: se necesita energía. Energía que no siempre tenemos.” (Gaia)*

*“En primer lugar, nuestra vida es muy ajetreada y agotadora. Todo gira en torno a Eduardo. Él requiere atención todo el tiempo. No hay forma de salir y dejarlo con cualquier persona. No habla, no camina, no se le puede dejar con un vecino o con un nieto, aunque la gente quiera ayudar. Conocemos cada señal que da, sabemos cómo es él. (...) Helena tiene la salud debilitada, ahora está de baja, pero aun así ayuda en todo lo que puede. Yo, cuando no estoy con Eduardo, trato de conseguir algún trabajo extra. Los hijos ayudan, pero cada uno tiene su vida. Así que nuestra vida es aquí dentro, con el niño, con los medicamentos, con los horarios del centro de salud, de la APAE y de las consultas.” (Atlas)*

Se observa que, ante fallas en la coordinación de los servicios o cambios en las condiciones de cuidado, las familias se ven a menudo obligadas a reconfigurar sus estrategias, ajustando rutinas, activando nuevas redes y redefiniendo prioridades. Estos movimientos evidencian que las condiciones intervinientes no actúan de manera estática, sino que se reinterpretan y se enfrentan continuamente en la vida cotidiana, lo que influye directamente en la forma en que se producen y sostienen las normas de cuidado inclusivo.

### El Trípode de las Invenciones Normativas en Acción (Estrategias de Acción/interacción)

Los datos indican que la invención y el mantenimiento de las normas de cuidado inclusivo se concretan a través de tres estrategias de acción/interacción interdependientes: coordinar rutinas (“planes vivos”), activar redes y *advocacy*, y poner en práctica la filiación. A partir de los testimonios de los participantes, se observa que estas estrategias no operan de manera aislada, sino que se articulan continuamente en la vida cotidiana, conformando un arreglo dinámico que sustenta la organización del cuidado frente a las demandas del niño y las condiciones del contexto.

*“Regresamos a nuestra ciudad, con todo ese cuidado que requería un niño con problemas respiratorios; vivíamos en un buen barrio de la ciudad, así que había buena calidad del aire, la gente nos visitaba de manera controlada (...) hacía fisioterapia para recuperarse del tiempo que pasó hospitalizado, hacía la fisioterapia en casa (...). Cuando regresamos, buscamos a quien pudiera ayudarnos; conocíamos a su esposo, nos pusimos en contacto con él y ella logró concertar una cita para que atendieran a Curetas”. (Orfeu)*

*Pedimos una semana para pensarlo. Necesitamos hablar entre nosotros. Porque es un cambio muy grande respecto a lo que habíamos pensado. Así que nos sentaremos a hablarlo. Pero en ese momento fue definitivo cuando los vimos”. (Hermes)*

En lo que respecta a la coordinación de las rutinas, los datos muestran que las familias construyen esquemas de organización del tiempo y las actividades, articulando terapias, la escuela, los desplazamientos, los momentos de ocio y descanso. Se observa que esta coordinación implica anticipar las necesidades, elaborar alternativas ante imprevistos y realizar ajustes continuos en las prácticas de cuidado, lo que contribuye a reducir las interrupciones y a lograr una mayor previsibilidad en la vida cotidiana.

*“Un ejemplo muy claro ocurrió ayer, en una actividad en la escuela de mi hijo menor. Era una pequeña fiesta escolar: música a todo volumen, mucha gente, bastante estimulación sensorial, un horario diferente al habitual y la exigencia de que los niños hicieran presentaciones para los padres. Y entre las familias presentes se encontraba una amiga nuestra, que tiene dos hijos pequeños, autistas, con un diagnóstico reciente. Durante la actividad, esos dos niños entraron en una crisis de desorganización. Estaban visiblemente angustiados, desorientados, reaccionando al exceso de estímulos que el ambiente les imponía. Había mucho ruido, demasiada gente. Y ahí estaba esa mamá, completamente sola, tratando de contener, calmar y proteger a sus hijos sin apoyo, sin acogida, sin estrategias. Eso me conmovió profundamente. Porque ya hemos vivido eso con los Curetas. Ya hemos estado en ese lugar de desesperación, de impotencia, de soledad ante la crisis de nuestro hijo.” (Réia)*

A partir de los relatos, se observa que la capacidad de respuesta institucional varía entre los diferentes servicios y momentos del proceso de atención, lo que genera experiencias que oscilan entre la acogida y las barreras.

Esta variación exige que las familias desarrollen estrategias específicas para traducir las necesidades del niño a un lenguaje que los servicios puedan reconocer, a menudo mediado por documentos como dictámenes, informes y planes institucionales.

*“El proceso para conseguir estas terapias comenzó en el centro de salud de nuestro barrio. Llevé los primeros dictámenes (...) y solicité las derivaciones a través del SUS. Ahí fue donde comenzó la maratón. (...) La terapia ocupacional también llegó por derivación del SUS, pero no en mi ciudad. Conseguí un cupo en una ciudad vecina, en un centro afiliado, que atendió a Leandro durante casi seis meses, hasta que el contrato se rescindió por cuestiones burocráticas entre el municipio y el proveedor. Desde entonces, volvimos a depender de la APAE (...) La psicoterapia fue lo más difícil de conseguir. A través del SUS, no había profesionales especializados en autismo infantil disponibles. Me derivaron al CAPSij (...), pero como no encajaba en ese perfil de urgencia clínica, terminó siendo “desregulado”. (...) La verdad es que el acceso a las terapias a través del SUS existe, pero está lleno de obstáculos: listas de espera, discontinuidad en la atención, falta de profesionales especializados en autismo e incluso dificultades para desplazarse.” (Gaia)*

En lo que respecta a la activación de redes y la *advocacy*, los datos indican que las familias movilizan recursos formales e informales para hacer posible el cuidado, involucrando a familiares, profesionales, instituciones y colectivos. Esta movilización incluye tanto el establecimiento de relaciones de apoyo como la elaboración y circulación de los documentos necesarios para acceder a los servicios y derechos.

Los participantes señalan que la construcción y el mantenimiento de estas redes exigen una inversión continua, y son fundamentales para ampliar el alcance de las estrategias familiares y reducir las barreras institucionales. Se observa que la presencia de actores clave—como los profesionales que facilitan el acceso a los servicios—puede favorecer la continuidad del cuidado.

En lo que respecta a la construcción de la filiación, los datos muestran que las familias desarrollan, en la vida cotidiana, prácticas que afirman la pertenencia del niño más allá del vínculo jurídico formal. Esta estrategia se expresa en interacciones, narrativas y decisiones que posicionan al niño como sujeto de derechos, sustentando su integración en los diferentes contextos de cuidado.

*“Hago algunos trabajos como albañil, ayudante, lo que se presente, porque la pensión no alcanza para todo. [...]. Así que la semana gira mucho en torno a eso: comida, medicinas, consultas, trabajos ocasionales y cuidar a Héspero”. (Atlas)*

Se observa que estas prácticas no solo organizan el cuidado, sino que también generan reconocimiento y estabilidad simbólica, influyendo en las decisiones relacionadas con la priorización de actividades, el manejo de las exigencias institucionales y el mantenimiento del vínculo familiar.

Los datos indican que estas tres estrategias—coordinación de rutinas, activación de redes y performación de la filiación—operan de manera integrada, ajustándose continuamente en respuesta a las condiciones intervenientes y a los acontecimientos de la vida cotidiana. Esta articulación constituye el núcleo operativo del modelo teórico, explicando cómo las familias inventan y estabilizan normas de cuidado inclusivo en contextos marcados por la variabilidad y la incertidumbre.

### Cuando las Normas se Materializan: Transformaciones Observables (Consecuencias)

Los datos indican que, cuando las estrategias de coordinación de rutinas, activación de redes y representación de la filiación se articulan de manera más estable, producen transformaciones observables en el cuidado, que se expresan en cuatro dimensiones interrelacionadas: participación, previsibilidad, comodidad y continuidad. A partir de los testimonios de los participantes, se observa que estas dimensiones no operan de manera aislada, sino que se construyen progresivamente en la vida cotidiana de las familias.

*“Esta experiencia, más que convertirnos en padres de una niña con discapacidad, nos transformó como personas. Nos hizo replantearnos valores, revisar prejuicios y ver la infancia y la diversidad humana con mucha más profundidad y humanidad.” (Ártemis)*

En lo que respecta a la participación, los datos muestran una mayor presencia y compromiso del niño en diferentes contextos, como la escuela, las actividades de ocio y la convivencia social, aunque en distintos niveles. Se observa que esta ampliación está relacionada con la capacidad de las familias para adaptar sus rutinas y negociar las condiciones de inclusión en los servicios.

La previsibilidad, por su parte, se manifiesta en la reducción de las incertidumbres relacionadas con los horarios, los flujos de atención y la organización de las actividades, y se construye mediante la anticipación de las necesidades y la consolidación de rutinas más estables.

*“Empezamos a tener momentos en familia que incluían a todos—juegos, paseos, viajes cortos—, y eso fue estrechando los lazos. El día que vi a Tritón enseñándole a Jápeto a ensamblar las piezas de un juguete educativo fue simbólico para mí. Sin que nadie lo pidiera, sin que hubiera una cámara grabando ni un público. Fue puro, natural y lleno de cariño. Hoy no tengo dudas: son hermanos. No solo en el papel, sino en el vínculo, en el gesto, en la mirada”. (Cronos)*

En cuanto al bienestar, los participantes señalan la reducción de situaciones de sobrecarga sensorial y emocional, tanto para el niño como para la familia, asociada a adaptaciones en el entorno, la comunicación y la organización del cuidado. Por su parte, la continuidad del cuidado se observa en una coordinación más consistente entre los diferentes servicios y contextos, lo que permite trayectorias menos fragmentadas a lo largo del tiempo.

*“Hoy, nuestra red de apoyo está bien definida, aunque no sea extensa. Contamos con pocas personas a nuestro alrededor, pero confiables, y eso ha sido suficiente [...].” (Atena)*

A partir de los relatos, se observa que estas transformaciones no eliminan las dificultades que enfrentan las familias, pero indican la construcción de una mayor estabilidad en el cuidado, aunque sea provisional y sujeta a reconfiguraciones.

*“Al principio, cuando llegó a nuestra casa y asumimos su custodia, ni siquiera sabíamos que tenía derecho a esas terapias. Fue la trabajadora social del juzgado quien nos lo explicó. (...) El médico pidió derivaciones al neurólogo, al fonoaudiólogo y al fisioterapeuta. Nos dijeron que podría tardar un poco—y de hecho tardó. Para conseguir esas terapias, fue un proceso lleno de papeleo. Teníamos que llevar el informe médico, el comprobante de domicilio, el número de la prestación, una fotocopia de esto, una fotocopia de aquello. Hubo días en que fuimos tres veces al mismo lugar para poder completar todo.” (Atlas)*

A partir de los relatos, se observa que la capacidad de respuesta institucional varía entre los diferentes servicios y momentos del proceso de atención, lo que genera experiencias que oscilan entre la acogida y las barreras.

Esta variación exige a las familias la construcción de estrategias situadas para traducir las necesidades del niño a lenguajes reconocidos por los servicios, a menudo mediados por documentos como dictámenes, informes y planes institucionales.

Los datos también indican la presencia de efectos asociados a este proceso, como la intensificación del trabajo de coordinación del cuidado y la necesidad continua de lidiar con exigencias burocráticas e institucionales. Al mismo tiempo, se observa que la movilización de redes y el fortalecimiento del vínculo familiar pueden contribuir a la redistribución de responsabilidades y a la ampliación del apoyo disponible.

*“La relación entre nuestros hijos es, sin duda, una de las mayores pruebas de que el amor no depende de la biología [...]. No fue solo que ‘aceptaran’ a sus hermanos. Realmente se abrieron” (Cronos)*

Por último, los datos muestran que la estabilidad de estas transformaciones varía según las condiciones intervinientes y los contextos institucionales, y se ve constantemente puesta a prueba por acontecimientos cotidianos que exigen nuevos ajustes y reconfiguraciones en las normas construidas por las familias.

## DISCUSIÓN

Este estudio propone una teoría sustantiva que explica cómo y por qué las familias adoptivas de niños con discapacidad inventan normas de cuidado inclusivo para sustentar la vida cotidiana. Los resultados indican que este proceso se organiza a partir de la articulación de tres estrategias de acción/interacción—coordinación de rutinas (“planes vivos”), activación de redes y *advocacy*, y representación de la filiación—orientadas por una ética del cuidado y moduladas por las condiciones intervinientes presentes en los contextos institucionales y sociales.

Se observó que estas estrategias no operan de manera aislada, sino que constituyen un arreglo dinámico que permite a las familias responder a las demandas del niño en escenarios marcados por la fragmentación de los servicios y las discontinuidades en el cuidado, un aspecto ampliamente descrito en la literatura sobre el cuidado de niños con condiciones complejas y sus familias^(18–20)^. En este sentido, los hallazgos desplazan el enfoque de un análisis centrado únicamente en las barreras o los impactos, poniendo de manifiesto los mecanismos procesales mediante los cuales las familias generan participación, previsibilidad, comodidad y continuidad a lo largo del tiempo^(21,22)^.

A partir de los resultados, se comprende que las normas de atención inclusiva no se configuran como protocolos fijos, sino como arreglos situados, construidos en la interacción entre las necesidades singulares del niño y las exigencias institucionales, en consonancia con enfoques que destacan la centralidad de la coordinación de la atención y de las experiencias familiares en los sistemas de salud^(21,22)^. Esta dinámica puede interpretarse a la luz del interaccionismo simbólico, al poner de manifiesto procesos de definición de situaciones, negociación de roles y construcción de acciones conjuntas en el día a día de la atención^(20)^. En diálogo con ello, la perspectiva de Canguilhem contribuye a comprender estas normas como expresiones de la capacidad de establecer nuevas referencias de funcionamiento ante variaciones de lo “normal”^(14)^.

En el ámbito de la salud, especialmente en la atención primaria, estos hallazgos refuerzan el papel estratégico de la enfermería en la mediación entre las familias y los servicios, en la coordinación de la atención y en la construcción de respuestas más sensibles a las necesidades concretas de los niños y sus cuidadores, en consonancia con la evidencia que señala la importancia de prácticas integradas y centradas en la familia^(4,22)^.

Al concebir las normas de cuidado inclusivo como artefactos interaccionales, los resultados evidencian el trabajo continuo de traducción, negociación y ajuste que realizan las familias para articular diferentes escenarios de cuidado, como el hogar, la escuela, la salud y la asistencia social. Se observó que este proceso implica no solo la organización práctica de las rutinas, sino también la mediación entre las expectativas institucionales y las necesidades particulares del niño, lo que exige a las familias una participación activa en la coordinación del cuidado.

Los hallazgos indican que este trabajo se intensifica en contextos marcados por la fragmentación de los servicios y la ausencia de flujos integrados, en los que las familias asumen la función de conectar los puntos de la red y garantizar la continuidad del cuidado, un aspecto ya descrito en la literatura sobre las cargas administrativas y las experiencias de los usuarios en los sistemas de salud^(21)^. En este sentido, prácticas como las visitas domiciliarias calificadas, la elaboración de Proyectos Terapéuticos Singulares y el seguimiento longitudinal, aunque se reconocen como fundamentales, siguen mostrándose subutilizadas en la Atención Primaria, lo que limita la efectividad de las acciones y la posibilidad de construir respuestas más ajustadas al contexto familiar^(22,23)^.

A partir de los resultados, se observa que las normas establecidas por las familias funcionan como mecanismos prácticos para estabilizar la atención, lo que permite armonizar las rutinas, reducir las incertidumbres y mantener la continuidad de las acciones a lo largo del tiempo. Este fenómeno refuerza la evidencia que destaca la importancia central de la coordinación del cuidado y de la participación familiar para lograr mejores resultados en salud, especialmente en contextos de mayor complejidad^(4,21,22)^.

Los resultados ponen de manifiesto un trípode operativo— coordinación de rutinas (“planes vivos”), activación de redes y *advocacy*, y desempeño de la filiación—como mecanismo central para sostener el cuidado a lo largo del tiempo. Se observó que estas estrategias se articulan en la vida cotidiana de las familias, lo que les permite lidiar con múltiples demandas simultáneas y responder a contextos institucionales a menudo fragmentados, en consonancia con la evidencia que señala la importancia central de la coordinación de la atención y de las intervenciones centradas en la familia^(4,7,20,24)^.

A partir de los hallazgos, se identifica que la coordinación de rutinas funciona como un mecanismo organizador de la atención, reduciendo las interrupciones y aumentando la previsibilidad de las acciones, mientras que la activación de redes y la *defensa de derechos* amplía el acceso a recursos y servicios, contribuyendo a la continuidad de la atención. Estos resultados concuerdan con estudios que muestran que las familias de niños con afecciones complejas suelen asumir la responsabilidad de la gestión de la atención, facilitando el acceso y coordinando diferentes servicios a lo largo del proceso de atención^(22)^.

En lo que respecta a la construcción de la filiación, los datos indican que la construcción cotidiana del sentido de pertenencia desempeña un papel central en el sostenimiento de la atención, influyendo en las decisiones relacionadas con la priorización de actividades, el manejo de las exigencias institucionales y la organización de las rutinas familiares. Este aspecto amplía la comprensión del cuidado más allá de su dimensión técnica, acercándose a enfoques que destacan el papel de las relaciones familiares, el vínculo y la comunicación en la prestación de cuidados en salud^(4,24)^.

Se observó además que la articulación entre estas tres estrategias se rige por una ética del cuidado que actúa como referencia para la toma de decisiones en situaciones de conflicto entre las exigencias institucionales y las necesidades del niño, tal como ya se ha señalado en estudios que abordan el cuidado centrado en la familia y la necesidad de ajustes contextuales en las prácticas asistenciales^(4,19)^. En este sentido, el trípode operativo identificado en este estudio contribuye a explicar cómo las familias construyen respuestas situadas capaces de sustentar la participación, la previsibilidad, el confort y la continuidad en contextos de alta complejidad.

Desde el punto de vista interaccionista, los resultados evidencian que la creación de normas de cuidado inclusivo se construye a partir de procesos continuos de definición de situaciones, negociación de roles y construcción de acciones conjuntas entre familias, niños y profesionales. Se observó que, en el día a día del cuidado, las familias interpretan las exigencias institucionales, ajustan las expectativas y redefinen las responsabilidades, generando arreglos que permiten sostener la organización del cuidado en contextos marcados por la variabilidad y la incertidumbre.

A partir de los hallazgos, se identifica que estas interacciones están mediadas tanto por experiencias concretas—como el acceso a los servicios y la capacidad de respuesta institucional—como por la capacidad de las familias para traducir sus necesidades a lenguajes reconocidos por los sistemas de salud, educación y asistencia social. Este proceso influye directamente en quién tiene legitimidad para participar en las decisiones y en cómo se organiza el cuidado a lo largo del tiempo, en consonancia con estudios que destacan la dimensión relacional e interaccional de la coordinación del cuidado^(20)^.

Se observa además que la repetición de experiencias—ya sean exitosas o difíciles—contribuye a la estabilización de ciertas prácticas, lo que permite a las familias consolidar rutinas, ajustar estrategias y mantener normas construidas en la vida cotidiana. En este sentido, los resultados indican que la previsibilidad del cuidado no se deriva únicamente de los protocolos institucionales, sino de la capacidad de construir y mantener acuerdos situados en las interacciones entre los diferentes actores involucrados.

A partir de los resultados, la filosofía de Canguilhem contribuye a comprender las normas del cuidado inclusivo como expresiones de la capacidad de instituir nuevas referencias de funcionamiento ante variaciones de lo “normal”^(14)^. Se observó que, cuando las respuestas institucionales no son suficientes para satisfacer las necesidades del niño, las familias comienzan a crear sus propios arreglos—ajustando rutinas, reorganizando el entorno y redefiniendo prioridades—para sostener el cuidado en la vida cotidiana.

En este sentido, los hallazgos indican que dichas normas no constituyen desviaciones respecto a los estándares institucionales, sino formas legítimas de organización de la vida, producidas en la interfaz entre las condiciones concretas y las posibilidades de acción de las familias. Esta perspectiva permite comprender que la continuidad del cuidado no depende únicamente del cumplimiento de protocolos, sino de la capacidad de crear soluciones situadas que respondan a las demandas específicas de cada contexto, en consonancia con la noción de normatividad de la vida propuesta por Canguilhem^(14)^.

Se observa además que estos procesos de invención normativa se intensifican en escenarios marcados por discontinuidades en los servicios y exigencias institucionales poco flexibles, en los que las familias asumen un papel activo en la construcción de alternativas para sostener el cuidado^(25,26)^. De este modo, los resultados refuerzan la importancia de reconocer estas prácticas no solo como estrategias de adaptación, sino como producciones normativas que pueden orientar respuestas más sensibles y contextualizadas en los servicios de salud.

Este estudio presenta limitaciones que deben tenerse en cuenta al interpretar los hallazgos. Se trata de una investigación cualitativa, realizada con un número reducido de participantes y en un solo municipio, lo que no permite hacer generalizaciones, aunque favorece una comprensión más profunda del fenómeno en su contexto^(27–30)^. Además, el análisis se construyó desde la perspectiva de los cuidadores, sin incluir directamente la voz de los niños y adolescentes, lo que puede limitar la comprensión de dimensiones importantes del cuidado. Aun así, el uso de la *Grounded Theory* y el logro de la saturación teórica confieren solidez analítica a los resultados, lo que permite la construcción de un modelo explicativo sensible a las variaciones y condiciones del fenómeno^(9–11)^.

Los resultados de este estudio indican que la creación de normas de cuidado inclusivo constituye un proceso relacional, dinámico y situado, sustentado por la articulación entre estrategias familiares, condiciones intervinientes y contextos institucionales. Al explicitar los mecanismos procesales que organizan el cuidado en la vida cotidiana, el modelo propuesto contribuye a comprender cómo se producen la participación, la previsibilidad, el confort y la continuidad en contextos marcados por la fragmentación de los servicios y las exigencias institucionales.

### Consideraciones Finales

Este estudio contribuye a comprender cómo y por qué las familias adoptivas de niños con discapacidad inventan y estabilizan normas de cuidado inclusivo en la vida cotidiana. Al explicitar el papel de la coordinación de rutinas, la movilización de redes y la construcción de la filiación en el sostenimiento del cuidado, el modelo propuesto amplía el análisis de los mecanismos que producen participación, previsibilidad, comodidad y continuidad en contextos marcados por la fragmentación institucional.

En el campo de la enfermería, los hallazgos reorientan el papel del enfermero en la atención primaria de salud hacia la función de gestor de casos (Case Manager) y navegador en salud. La actuación de este profesional debe ir más allá de la atención clínica directa, centrándose en la coordinación proactiva de los flujos intersectoriales y en la mediación técnica entre las familias y la red de servicios (salud, educación y asistencia social).

La enfermería familiar, al identificar de manera temprana las brechas en la atención y la sobrecarga de los cuidadores, tiene el potencial de reducir la necesidad de que las familias “invente” normas por su cuenta, promoviendo la estabilización de la atención mediante planes terapéuticos integrados que garanticen la previsibilidad y la continuidad de la atención.

Se recomienda el desarrollo de estudios longitudinales, comparativos y de implementación, así como investigaciones que incorporen la perspectiva de los niños y adolescentes, con el fin de profundizar en la comprensión de los procesos de cuidado y de las condiciones que favorecen su sostenibilidad.

## Data Availability

El conjunto de datos que respaldan las conclusiones de este estudio está disponible previa solicitud al autor correspondiente.
